# Cascade Immune Mechanisms of Protection against *Mycobacterium tuberculosis* (IMPAc-TB): study protocol for the Household Contact Study in the Western Cape, South Africa

**DOI:** 10.1186/s12879-022-07349-8

**Published:** 2022-04-15

**Authors:** Andriёtte M. Hiemstra, Candice E. MacDonald, Ilana C. van Rensburg, Kim Stanley, Elizna Maasdorp, Shirley Mc Anda, Susanne Tönsing, Jane Alexandra Shaw, Gerard Tromp, Gian D. van der Spuy, Kevin B. Urdahl, David M. Lewinsohn, Helena Kuivaniemi, Nelita Du Plessis, Stephanus T. Malherbe, Gerhard Walzl

**Affiliations:** 1 Biomedical Research Institute Clinical Team, Stellenbosch University, Cape Town, South Africa; 2 Division of Molecular Biology and Human Genetics, Department of Biomedical Sciences, Stellenbosch University, Cape Town, South Africa; 3 DSI–NRF Centre of Excellence for Biomedical Tuberculosis Research, Stellenbosch University, Cape Town, South Africa; 4 South African Medical Research Council Centre for Tuberculosis Research, Stellenbosch University, Cape Town, South Africa; 5 South African Tuberculosis Bioinformatics Initiative, Stellenbosch University, Cape Town, South Africa; 6 Centre for Bioinformatics and Computational Biology, Stellenbosch University, Stellenbosch, South Africa; 7 Seattle Children’s Research Institute, Seattle, USA; 8 Departments of Pediatrics and Immunology, University of Washington School of Medicine, Washington, USA; 9 Division of Pulmonary and Critical Care Medicine, Department of Medicine, Oregon Health and Science University, Portland, USA

**Keywords:** Tuberculosis, Immune response, Bronchoalveolar lavage, PET-CT imaging, Household contacts, SARS-CoV-2, Interferon-gamma release assay

## Abstract

**Background:**

Natural immunity against *Mycobacterium tuberculosis* exists, and > 90% of those infected remain disease-free. Innate and adaptive immune responses required to mediate such protection against tuberculosis (TB) are, however, poorly understood.

**Methods:**

This is an analytical study exploring protective and non-protective pathways of immunity against *Mycobacterium tuberculosis*. Adults without HIV infection are recruited at community healthcare clinics in high TB incidence areas of the Western Cape Province, South Africa. Data regarding participants’ medical, social and medication usage will be collected, and clinical examinations and point-of-care tests documented. Reference tests for TB (chest radiographs and sputum tests for GeneXpert MTB/RIF Ultra®, Auramine smear and liquid cultures) and investigations to classify infection states [interferon-gamma release assay (IGRA) and SARS-CoV-2 polymerase chain reaction (PCR) nasopharyngeal swab and IgG], are done on all participants who meet the inclusion criteria. 18F-Fluorodeoxyglucose positron emission tomography combined with computerized tomography will be done on all close contacts (contacts) and healthy control (controls) participants. Participants are divided into 12 study groups representing a spectrum of TB clinical phenotypes and prior SARS-CoV-2 infection based on their TB status, exposure history, results of IGRA test at baseline and 3 months, SARS-CoV-2 serology, and PCR results, and for contacts and controls, PET-CT imaging findings indicative of sub-clinical TB lesions. Samples for experimental assays include whole blood for isolation of peripheral blood mononuclear cells and blood in PAXgene® tubes for RNA isolation. All SARS-CoV-2 PCR negative study participants undergo bronchoscopy for collecting bronchoalveolar lavage samples.

**Discussion:**

The paired blood and BAL samples will be used for comprehensive analyses of the tissue-specific and systemic immunity that will include e.g., cytometry by time-of-flight analyses, RNA-sequencing, multiplex immunoassays, epigenetic analysis, and mechanistic studies of control of infection by *Mycobacterium tuberculosis*. Results will be integrated with those from mice and non-human primate studies to provide a comprehensive analysis of protective pathways in natural and vaccine-induced immunity against *Mycobacterium tuberculosis*.

**Supplementary Information:**

The online version contains supplementary material available at 10.1186/s12879-022-07349-8.

## Background

Despite major advances in diagnosis, treatment, and prevention during the last century, tuberculosis (TB) is still a leading infectious cause of death globally and killed 1.4 million people in 2019 alone [1, 2]. Bacille Calmette–Guérin (BCG) vaccine, introduced in 1921, protects the young against severe forms of TB but varies in effectiveness in adults and other forms of TB [3]. The emergence of drug-resistant strains of TB further heightens the need for more effective vaccines. To develop and evaluate novel strategies in vaccine development and host-directed therapy, it is crucial to understand host immune responses against *Mycobacterium tuberculosis* (Mtb). These mechanisms exist, as more than 90% of those infected remain disease-free [4], but they are poorly understood. Elucidating the immune mechanisms that underlie this resistance could open new avenues for the design and early evaluation of more effective vaccines.

Several factors limit understanding of the complex natural protection against TB. Protection can occur at various stages of infection and likely ranges from pathogen containment to clearance [4]. It is now well-established that some household or close contacts of an index case with active TB, who initially convert to a positive (QFT TB-Nil 1 and 2 values > 0.35) interferon-gamma release assay (IGRA), an indication of an Mtb-specific T cell response that reflects infection, will later revert to a negative IGRA (QFT TB-Nil 1 and 2 values < 0.35) [5]. Additionally, prior Mtb infection is a major driver of natural immunity, but it is difficult to definitively identify protected and unprotected individuals in high transmission settings [5]. Protective immunity is probably established in the first days to few weeks after aerosol Mtb exposure, but infected individuals are usually not identified until many weeks to months later. Lastly, protective immune responses occur at tissue sites of infection, which are generally inaccessible for sample collection. Human immune responses are usually assessed in the blood, and their correlation to the tissue site responses is poorly understood.

To overcome these barriers to understanding human natural and vaccine-induced immunity to TB, a multi-disciplinary team of experts using advanced microscopy, systems biology, and computational modelling, will conduct a comprehensive, multisite, international study called Cascade Immune Mechanisms of Protection against *Mycobacterium tuberculosis* (IMPAc-TB). This study is funded by the National Institutes of Health (NIH), USA and is led by the Seattle’s Children Research Institute (SCRI) in the US. The main goal of Cascade IMPAc-TB is to inform the rational design of an effective TB vaccine by fundamentally expanding our insights into immune responses that can control and potentially even eradicate Mtb. This goal will be achieved by identifying and dissecting protective pathways in infection- and vaccine-induced immunity through integrated analyses of tissue-specific and systemic immunity in mice, non-human primates (NHP), and humans. Here we describe the design and clinical procedures of the Cascade Household Contact Study to be carried out by the Stellenbosch University (SU) investigators in the Western Cape Province, South Africa. In this study, an in-depth analysis of innate and adaptive immune responses in the peripheral blood and lungs will be performed.

## Methods

### Aims and objectives

The primary goal of the Cascade Household Contact study is to carry out a comprehensive clinical assessment and collect biological samples from study participants representing a spectrum of TB clinical phenotypes that are defined by 18F-Fluorodeoxyglucose (FDG) positron emission tomography-computed tomography (PET-CT) and immunologic criteria. Classification of the participants into different phenotypic groups will allow results to be compared between groups on different analytical platforms. Participants will also be classified according to evidence of other respiratory infections, including Severe Acute Respiratory Syndrome Coronavirus 2 (SARS-CoV-2) and other common respiratory viruses. Samples from those who have had previous SARS-CoV-2 infection will be analysed for any potential impact on host immune responses to Mtb.

### Design and setting of the study

The Cascade Household Contact Study is an observational, case–control, analytical study on human subjects.

Study participants will be enrolled from several areas in Cape Town, Paarl, and the West Coast region in the Western Cape Province, South Africa. These communities carry a high burden of TB disease, up to 880/100,000 population in some areas, and are characterized by poverty, overcrowding and limited health care resources.

The Stellenbosch University Immunology Research Group (SU-IRG) together with the Biomedical Research Institute Clinical Team (BMRI CT) will be responsible for all study participant recruitment and sample collection. The blood and bronchoalveolar lavage (BAL) samples collected will be used for comprehensive analyses of the tissue-specific and systemic immunity that will include cytometry by time-of-flight analyses (CyTOF), RNA-sequencing (RNA-seq), and multiplex immunoassays, epigenetic analysis, and mechanistic studies of Mtb control. Some aliquots of the samples collected through phlebotomy and BAL will be analysed at Stellenbosch University, whereas others will be shipped to partners of Cascade IMPAc-TB project at Oregon Health and Science University (OHSU), SCRI, and the University of Washington (UW) for further analyses (to be described elsewhere).

### Participant recruitment and study groups

A Community Advisory Board (CAB) consisting of members of the communities from which participants are recruited meets once a month. The board functions as a forum to advocate for human rights and ethical treatment of research study participants and may contribute to addressing grievances about the research process, give advice on recruitment and retention of study participants, and voice community concerns about study procedures or outcomes. All protocols and informed consent documents are reviewed by the board before implementation. The members of the board also help raise awareness of TB and research in the community.

**Fig. 1:**
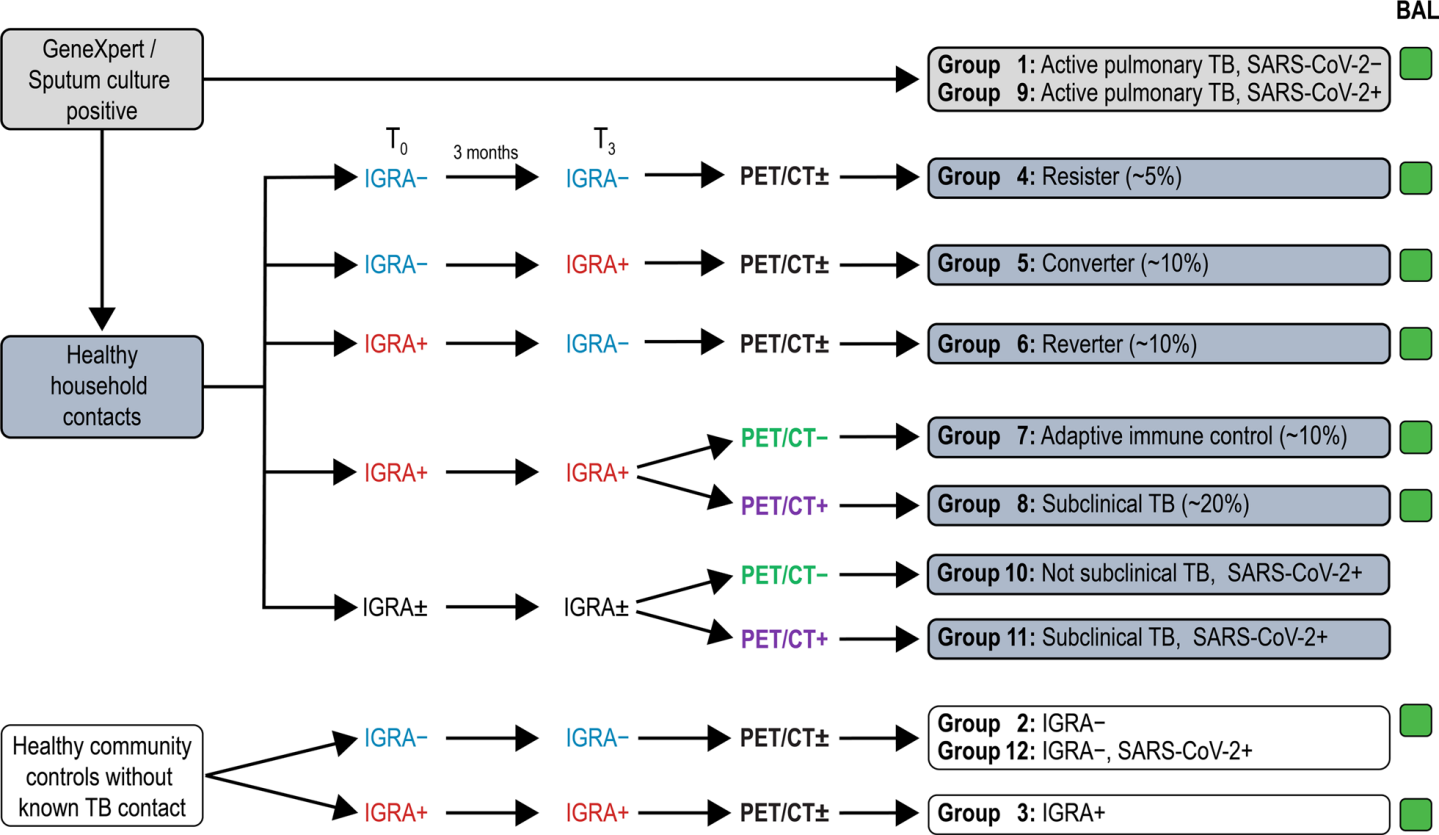
Study groups. This is summary of all 12 groups which are part of the Cascade IMPAcTB HHC study at Stellenbosch University

A total of 236 HIV negative adult study participants with no previous history or serological evidence of SARS-CoV-2 infection (20 subjects for groups 1–8 described in Fig. 1 plus 76 additional participants for follow-up studies) will be recruited and enrolled. TB-index cases will be identified from clinics in and around Cape Town, South Africa with the help of community-based research workers (CRW), and with their consent, their contacts will be approached.

In addition, a total of 80 participants with evidence of previous SARS-CoV-2 infection will be recruited to the study for groups 9–12 (Fig. 1). These participants will be identified through a combination of passive recruitment from amongst those who test positive in other ongoing clinical studies, and active recruitment from the local testing center at Tygerberg Academic Hospital and the National Health Laboratory Services. A previous positive SARS-CoV-2 polymerase chain reaction (PCR) or current positive SARS-CoV-2 serology test will be considered evidence of previous SARS-CoV-2 infection. Testing for SARS-CoV-2 antibodies will be done at screening and repeated within 7 days before bronchoscopy is done in contacts and controls. As vaccination rates increase in the population, it is anticipated that vaccine-induced antibodies will be detected in a growing number of participants and thus the month 3 SARS-CoV-2 antibody tests will be done with both anti-nucleocapsid antibodies (Architect® SARS-CoV-2 IgG, Abbott Laboratories, Sligo, Ireland) and anti-spike protein antibodies (Architect® SARS-CoV-2 IgG II Quant, Abbott Laboratories, Sligo, Ireland). Interpretation of these results combined with history of infection and/or immunization to determine inclusion into the SARS-CoV-2 positive group, will be done as summarized in Table 1. SARS-CoV-2 Real-Time PCR tests on nasopharyngeal swabs will only be done within 7 days before bronchoscopy for infection transmission prevention.

**Table 1:** Interpretation of SARS-CoV-2 antibody serology

	Anti-N ab positive, unvaccinated	Anti-N ab positive, vaccinated	Anti-N ab negative, vaccinated	Anti-N ab negative, unvaccinated
Anti-S protein ab positive	Infection induced immunity: SARS-CoV-2 positive group	Both infection-induced immunity and vaccine response, or waned vaccine response and both antibodies due to infection induced immunity: SARS-CoV-2 positive group	Vaccine-induced immunity: SARS-CoV-2 negative group	Infection induced-immunity: SARS-CoV-2 positive group
Anti-S protein ab negative	Infection induced immunity: SARS-CoV-2 positive group	Vaccine induced immunity has waned below detectable level, and breakthrough infection has occurred: SARS-CoV-2 positive group	SARS-CoV-2 negative group	SARS-CoV-2 negative group

Participants must fulfil all the inclusion and exclusion criteria to be eligible for enrolment (Table 2).

**Table 2:** Inclusion and exclusion criteria

Inclusion criteria		
A. All participants		
• 18–65 years old		
• Weight > 35 kg and < 120 kg		
• HIV negative		
• Willing and able to comply with study procedures		
• No known current systemic infections (apart from active TB for group 1)		
• Agree to have a bronchoscopy performed		
• Agree to undergo a PET-CT		
• Agree to have a chest x-ray done		
• Have a verifiable address		
B1. TB cases (A + B1)	B2. Contacts (A + B2)	B3. Controls (A + B3)
• No history of previous active TB within the last 3 years (3 years since completion of TB treatment) • TB diagnosis confirmed by the following: Smear positive OR GeneXpert® positive medium, high, or very high OR GeneXpert® positive lower than medium (i.e., trace, very low or low positive) plus symptoms suggestive of active PTB or a chest x-ray suggestive of PTB	• Contacts of newly diagnosed active pulmonary TB patients or participants who have shared the same home, work, or recreational space as the index case for at least 3 months prior to TB diagnosis and for at least 5 h per week in the same room • Negative GeneXpert® • No symptoms or signs suggestive of active TB—unintentional weight loss, drenching night sweats, coughing for 2 or more weeks, hemoptysis	• Member of the community who does not have close contact to a patient or participant with active pulmonary TB and who self does not have active TB • Negative GeneXpert® • No symptoms or signs suggestive of active TB—unintentional weight loss, drenching night sweats, coughing for 2 or more weeks or hemoptysis
C. SARS-CoV-2 positive groups (A + B1/B2/B3 + C)		
• Participants who test positive for SARS-CoV-2 on a validated serologic test, regardless of previous symptoms		
• Participants who have previously tested positive for SARS-CoV-2 on respiratory specimen PCR or antigen testing, as per the Western Cape Provincial case definition		

Exclusion criteria		
A. All participants		
• Pregnant (current or in the last 6 months) or breastfeeding		
• No permanent address		
• Hb < 9 g/dl		
• HIV infection		
• Planned relocation or pending criminal cases potentially leading to incarceration during the duration of the study		
• Diabetes mellitus as defined by random glucose ≥ 11.1 mmol/l (or ≥ 200 mg/dl), fasting plasma glucose ≥ 7.0 mmol/l (≥ 126 mg/dl), point of care HbA1c ≥ 6.5, or the use of any anti-diabetic agent (including traditional medicines) as a concomitant medicine		
• Use of any immunosuppressive drugs within the last 4 weeks		
• Any severe systemic condition/co-morbidity that may affect the safety of the participant or the performance of the assays, including (but not limited to) uncontrolled diabetes, cancer, uncontrolled hypertension, or ischemic heart disease		
• Any person for whom the physician/study nurse feels this study is not appropriate for, or that the study is not in the person’s best interest, e.g., a history of substance or alcohol abuse that may interfere with the participant’s adherence to study procedures		
• History of taking any TB medication in the past 4 weeks		
• Known allergy or intolerance to components of FDG or drugs used in procedural sedation		
• A known history or family history of malignant hyperthermia		
B1. TB positive cases (A + B1)	B2. Contacts (A + B2)	B3. Controls (A + B3)
• Already started TB treatment • Resistance of TB strain to TB medication • Evidence of or suspected extra-pulmonary TB (based on clinical judgement or confirmed by microbiological studies or radiologically) Participants with a TB pleural effusion will be included provided that the effusion is small to moderate in size and there is overt evidence of parenchymal involvement on the chest radiograph • History of previous active Mtb disease/TB treatment within the last 3 years	• Previous active Mtb disease • Any microbiological evidence of current active TB disease (positive GeneXpert®/smear/culture)	• Previous active Mtb disease • Any microbiological evidence of current active TB disease (positive GeneXpert®/smear/culture) • History of contact with a patient with active TB in the last 12 months • Chest X-ray evidence of chronic lung disease or active TB

*FDG* fluorodeoxyglucose; *Mtb Mycobacterium tuberculosis*; *PTB* pulmonary tuberculosis; *TB* tuberculosis

Participants will first be stratified into the following four *arms* (Fig. 1):

- Participants with pulmonary TB (cases)
- Healthy Community Controls (controls)
- IGRA-negative contacts, or
- IGRA-positive contacts

Further classification of contacts and controls within these arms will be based on two IGRA (QuantiFERON-TB Gold Plus® (QFT) assay, Qiagen, Carnegie, Australia) results, performed 3 months apart, as well as the outcomes of PET-CT done within 7 days of the month 3 IGRA test. A combination of TB minus nil 1 and TB minus nil 2 values are used to interpret the QFT test. Should both values be > 0.5 IU/ml, the participant will be classified as IGRA positive. Should both values be < 0.3 IU/mI, the participant will be classified as IGRA negative (< 0.3 IU/ml). If both values are between 0.3 and 0.5 IU/ml or one value is < 0.3 IU/ml and the other between 0.3 and 0.5 IU/ml, the participant will be excluded due to QFN values in the “grey zone”. In cases where one value is equal to or above 0.5 and the other between 0.3 and 0.5, the participant will be included as IGRA positive.

**Fig. 2:**
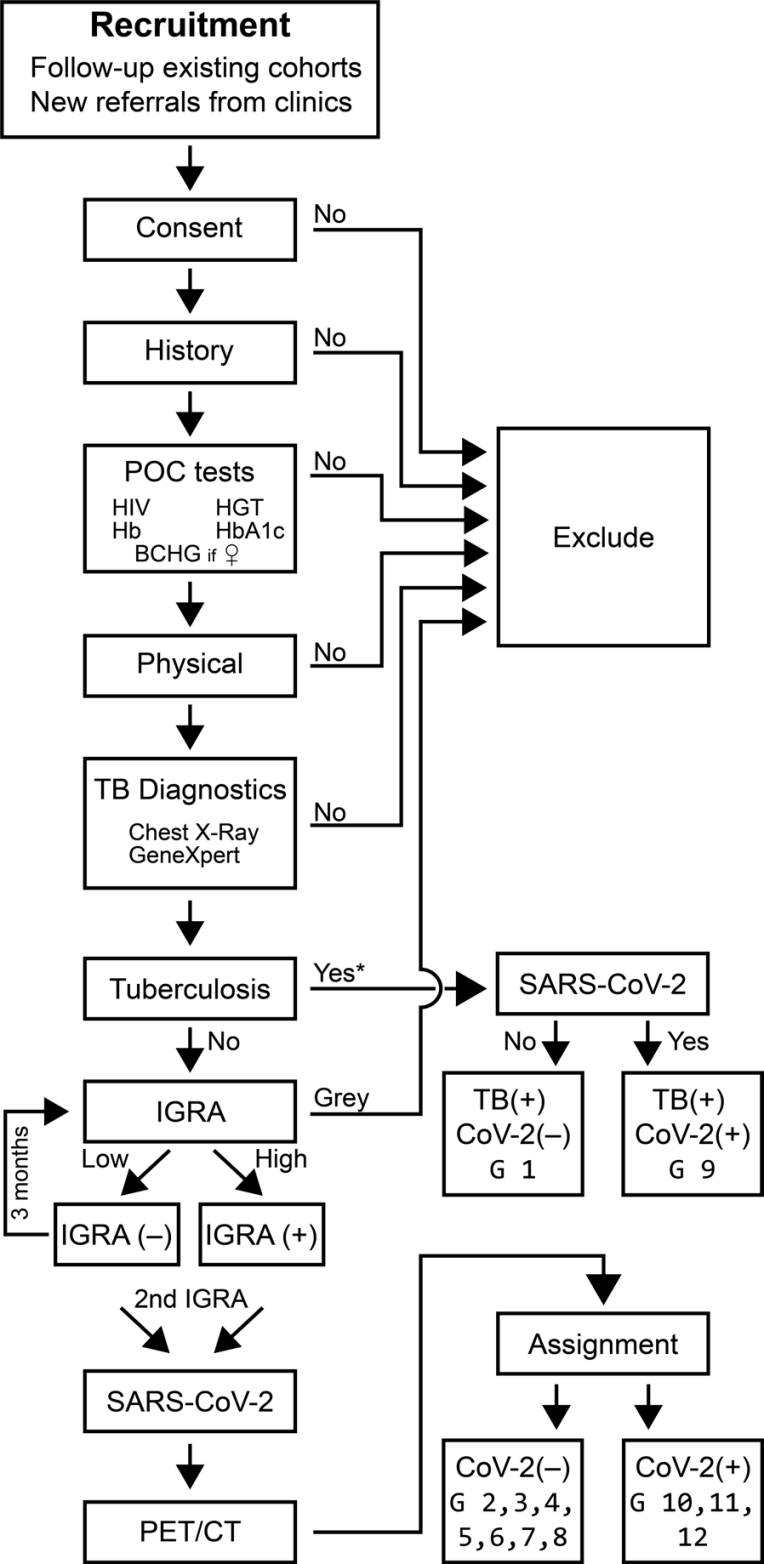
Summary of screening and enrolment procedures. All contacts and control participants who are eligible to be enrolled into Cascade Household Contact study will undergo PET-CT imaging of their lungs. In addition, all eligible SARS-CoV-2-negative participants will undergo bronchoscopy in which bronchoalveolar lavage (BAL) samples are collected

The final classification will further divide the cohort into the following groups (Figs. 1 and 2):

1. Active untreated pulmonary TB cases: These participants will have newly diagnosed, drug-sensitive, active pulmonary TB (PTB) but will not be on treatment yet. TB cases will be confirmed by one of the following: Positive Auramine smear or GeneXpert® MTB/RIF Ultra (Cepheid, Sunnyvale, USA) positive medium, high, or very high or GeneXpert® MTB/RIF Ultra positive lower than medium (i.e., trace, very low or low positive) plus symptoms suggestive of active PTB or a chest x-ray suggestive of PTB. Those with HIV infection (diagnosed by Rapid Point of Care test, U-Test HIV/AIDS, Humor Diagnostica, Guangzhou, China) and confirmed twice, will be excluded.
2. IGRA-negative healthy community controls: Individuals in this group will have a negative QFT (TB antigen minus nil value < 0.3 IU/ml) at screening and month 3. Those with discordant results between screening and month 3, will be excluded from the study, as will those with any of the QFT TB minus nil values in the “grey zone” between 0.3 and 0.5 IU/ml.
3. IGRA-positive healthy community controls: This group will have positive QFT (TB minus nil tests values > 0.5 IU/ml) at screening and month 3. This group presumably has stable latent TB infection and is expected to represent around 75% of the individuals living in this high TB prevalence community. Active TB will be excluded with a symptom screen, chest radiograph, and sputum GeneXpert MTB/RIF Ultra®. Two consecutive QFT tests will be done at screening and month 3 visits and those community controls with discrepant results will be excluded.
4. IGRA-negative contacts with a subsequent negative IGRA (Resisters): These participants represent a group with high resistance to Mtb infection as they have reached adult life in a high transmission setting without becoming QFT-positive and have subsequently also endured potential exposure to an active TB case in their household without becoming sensitized. Again, only those with two negative QFT tests (TB minus nil < 0.3 IU/ml) will be included in this group.
5. IGRA negative contacts with a subsequent positive IGRA (Converters): These participants initially have a negative IGRA result but subsequently convert to a positive result. To rule out fluctuations occurring by chance between positive and negative results, all participants with QFT TB minus nil values between 0.3 and 0.5 IU/ml will be excluded. These IGRA converting participants may represent a group with recent Mtb infection that controls infection through adaptive immune responses.
6. IGRA positive contacts with a subsequent negative IGRA (Reverters): These contacts initially have a positive QFT but become test-negative within 3 months. They might represent a protected phenotype as they do not appear to have a stable infection, as indicated by IGRA positivity. To ensure that we are not merely dealing with test fluctuations close to the test positivity cut-off, we will only regard changes as reversions if TB minus nil values > 0.5 change to < 0.3 IU/ml.
7. IGRA positive contacts who remain IGRA-positive on repeat testing and have a negative PET-CT (adaptive immune control): Most IGRA-positive individuals will have negative PET-CT scans. This group represents individuals in whom adaptive immune responses have presumably contained infection to a passive state, possibly without eradication of bacteria.
8. IGRA positive contacts who remain IGRA positive on repeat testing and have a positive PET-CT (subclinical disease): We anticipate that 25% of recent contacts will have FDG-avid lesions in PET imaging [6]. Active TB will be excluded with a symptom screen, chest radiograph, and sputum GeneXpert MTB/RIF Ultra®. These participants represent a group in which adaptive immune responses contain Mtb infection to a subclinical state of replication. This group will be followed up by study staff for 2 years or to study completion to capture potential progression to active TB.
9. Active untreated pulmonary TB cases with positive SARS-CoV-2 serology test: This group will be analogous to Group 1 above, but the participants will test positive for recent SARS-CoV-2 infection when a serology-based test is used.
10. Contacts with stable IGRA and negative PET-CT with positive SARS-CoV-2 serology or PCR test: This group will be analogous to Group 7 above, but the participants will test positive for active or recent SARS-CoV-2 infection when either serology or a nasopharyngeal swab PCR-based test is used.
11. Contacts with stable IGRA and positive PET-CT with positive SARS-CoV-2 serology or PCR test: This group will be analogous to Group 8 above, but the participants will test positive for active or recent SARS-CoV-2 infection when either serology or a nasopharyngeal swab PCR-based test is used.
12. Controls with stable IGRA test results and positive SARS-CoV-2 serology or PCR test (irrespective of PET-CT): This group will be analogous to Groups 2 and 3 above, but the participants will test positive for active or recent SARS-CoV-2 infection when either serology or a nasopharyngeal swab PCR-based test is used.

### Assessments: clinical procedures (see summary Fig. 2)

Individuals indicating interest in participating in the study will be pre-screened by CRWs using an online REDCap (Research Electronic Data Capture) [7, 8] based screening tool designed to ascertain suitability for screening. Potentially eligible participants will then be transported to the study site on the Tygerberg Medical Campus of SU. Informed written consent will be obtained before any study procedures are undertaken. All further evaluations will be conducted by the BMRI CT at the Tygerberg Medical Campus and will be scheduled according to the designated arm for the participant.

Demographic data along with participants’ medical, surgical, medication and drug usage history will be collected, and clinical examinations and point-of-care tests documented. All participants will have point of care tests for hemoglobin, random blood glucose and HIV. Females of childbearing potential will also have a urine β-hCG pregnancy test (Homemed, Assure Tech, Hangzhou, China). For participants with a random blood glucose of ≥ 7 mmol/l, a point-of-care HbA1c will be done to exclude Diabetes Mellitus. All participants will receive pre-and post-counselling for HIV tests and positive tests will be confirmed with a second rapid test. All discrepant tests or twice positive tests will be confirmed by a Western Blot Elisa HIV test. Participants excluded due to a new diagnosis of HIV or pregnancy will be referred to the appropriate community healthcare facility for further management.

Sputum, blood, and BAL will be collected from cases, controls, and contacts. This includes those with evidence of sub-clinical infection based on PET-CT. Contacts will be further categorized by their different immunologic response to Mtb antigens over 3 months (indicative of past or present Mtb infection), into those persistently IGRA-negative, persistently IGRA-positive, IGRA-converters (IGRA-negative to IGRA-positive, indicating recent infection), and IGRA-reverters (IGRA-positive to IGRA-negative, suggesting possible clearance). These groups will be compared to one another and controls. In each group, lung immune responses will be linked to blood transcriptional signatures and a comprehensive phenotypic and functional analysis of individual blood immune cells, including Mtb antigen-specific T cells and innate cells.

All participants will be assessed for current or previous SARS-CoV-2 infection, and a group of participants who test positive will be used to assess the potential impact of recent SARS-CoV-2 infection on results.

#### Active pulmonary TB cases (visits and procedures summarized in Table 3)

**Table 3:** Visiting schedule and procedures for TB participants (Cases)

Study day	Screening and enrolment	Visit 1	Contact 1
	Day 0	Day 1–5 (no treatment) (Bronchoscopy)	72 h and day 14 post bronchoscopy call
Informed consent including bronchoscopy consent and genetic testing consent	x		
Inclusion/exclusion criteria	x		
Targeted physical examination	x	x	
Vital signs and medical history	x	x	
Concomitant medications	x		
Chest X-ray	x		
ECG (If clinically indicated)		x	
Finger prick blood (Hb, random glucose, HbA1c, if indicated)	x		
HIV-1 rapid counselling and test	x		
Sputum (for GeneXpert®, smear & MGIT)	x		
PAXgene®		x	
Urine βHCG (all females)	x		
NaHep (PBMC)	x	x	
Bronchoscopy (BAL)		x^a^	
Serum	x (SARS-CoV-2 serology)	x	
Nasopharyngeal swab (for SARS-CoV-2 PCR & respiratory pathogen assays)	x		
Phone call and symptom check			x

^a^Only if SARS-CoV-2 PCR negative

Participants with GeneXpert® MTB/RIF Ultra confirmed active PTB not yet on TB treatment, will be approached by study personnel at the community clinics to determine interest in study participation. All study procedures will be completed within 5 days of the screening visit, when these individuals will be referred to the community clinic for initiation of the standard of care TB treatment. Participants recruited as contacts or controls diagnosed with active PTB during the screening of participants in other observational studies conducted by the BMRI Clinical Team newly diagnosed with active PTB may also be included in this group should inclusion and exclusion criteria be met.

#### Controls (visits and procedures summarized in Table 4)

**Table 4:** Visiting schedule and procedures for contacts

	Screening and enrolment	Visit 1	Visit 2	Visit 3	Contact 1	3-monthly follow up call (Group 8 only)
Study day	Day 0	M 3–7 days	M3 PET-CT	M3 + 7–14 days (Bronchoscopy)	72 h and day 14 post bronchoscopy	Telephonic/home visit follow up
Informed consent including bronchoscopy, PET-CT, and genetic testing	x					
Inclusion/exclusion criteria	x					
Targeted physical examination	x			x		x^d^
TB symptom screen	x	x				x
Vital signs and medical history	x		x	x		
Concomitant medications	x					
Chest x-ray	x					x^d^
ECG (If clinically indicated)				x		
IGRA	x	x				
Finger prick blood (Hb, random glucose, HbA1c, if indicated)	x		x^b^			
HIV-1 test and counselling	x					
Sputum (GeneXpert® & MGIT)	x	x^a^				x^d^
PAXgene®				x		
Urine βHCG (all females)	x		x	x^c^		
NaHep (PBMC)			x	x		
Bronchoscopy (BAL)				x^e^		
Serum	x (SARS-CoV-2 serology)	x (storage)	x (SARS-CoV-2 serology)	x		
Nasopharyngeal Swab (for SARS-CoV-2 PCR & respiratory pathogen assays)			x			
PET-CT			x			
Phone call and symptom check					x	

^a^GeneXpert® only for participants with new TB symptoms

^b^Only POC fasting glucose on PET-CT visit

^c^If not done within the last 14 days

^d^Only if TB symptom screen positive

^e^Participants testing SARS CoV 2 PCR positive at visit 2 will have bronchoscopy delayed until 14 days after clinical recovery or PCR negative test, whichever is longer

Healthy people accompanying relatives or friends to the clinic or members of the community referred from elsewhere, who fit all the inclusion and exclusion criteria will be recruited.

#### Contacts (visits and procedures summarized in Table 5)

**Table 5:** Visiting schedule and procedures for controls

	Screening and enrolment	Visit 1	Visit 2	Visit 3	Contact 1
Study day	Day 0	M3 − 7 days	M3	M3 + day 7–14 (bronchoscopy)	72 h and day 14 post bronchoscopy
Informed consent including bronchoscopy, PET-CT, and genetic testing	x				
Inclusion/exclusion criteria	x				
Targeted physical examination	x			x	
TB symptom screen	x	x			
Vital signs and medical history	x		x	x	
Concomitant medications	x		x		
Chest X-ray	x				
ECG (If clinically indicated)				x	
Finger prick blood (Hb, random glucose, HbA1c, if indicated)	x				
HIV-1 test and counselling	x				
IGRA	x	x			
Sputum (GeneXpert® & MGIT)	x	x^a^			
PAXgene®				x	
Urine βHCG (all females)	x		x		
NaHep (PBMC)			x	x	
Bronchoscopy (BAL)				x^b^	
PET-CT			x		
Serum	x (SARS-CoV-2 serology)		x (SARS-CoV-2 serology)	x	
Nasopharyngeal swab (for SARS-CoV-2 PCR & Respiratory pathogen)			x		
Phone call and symptom check					x

^a^GeneXpert only for participants with new TB symptoms

^b^Participants testing SARS-CoV-2 PCR positive at visit 2 will have bronchoscopy delayed until 14 days after clinical recovery or PCR test, whichever is longer

Participants recently diagnosed with active PTB at clinics or as part of the BMRI Clinical Team’s ongoing studies and clinical trials will be approached and asked if they are willing to share contact details of their relatives, co-workers, or others whom they meet regularly. Those contacts who have shared the same home, work, or recreational space as the index case for at least 3 months before TB diagnosis and at least 5 h per week in the same room will be contacted by CRWs or visited at home to determine interest in the study. Interested individuals will be screened for eligibility.

### Blood sampling

Blood sampling will take place at the study site according to the schedule for the specific group allocation for the participant summarized in Tables 3, 4 and 5. Following the guidelines set out in Table 6, no more than 140 ml of whole blood will be collected within 3 months. For TB patients, no more than 130 ml of whole blood will be collected within 5 days.

**Table 6:** Blood volume collection per visit

Group	Visit	Max volume (ml)
TB participants		
	Screening (D0)	60
	Bronchoscopy (D1–5)	70
Contacts		
	Screening (D0)	4
	Visit 1 (M3 – 7 days)	4
	Visit 2 (M3)	60
	Visit 3 (M3 + 7–14 days)	70
Controls		
	Screening (D0)	4
	Visit 1 (M3 – 7 days)	4
	Visit 2 (M3)	60
	Visit 2 (M3 + 7–14 days)	70

Peripheral blood mononuclear cells (PBMCs) will be isolated and cryopreserved by SUN-IRG for further assays. Proposed assays will be conducted on unfractionated PBMC as well as selected sorted cell populations and analyzed at SU, OHSU, SCRI, UW and other partners. Assessments will include phenotyping by CyTOF or flow cytometry, limited dilution T cell cloning, T-cell receptor (TCR) sequencing, single-cell or bulk RNAseq, multiplex immunoassays and functional granuloma assays to assess differences (e.g., cytokine production, antimicrobial function) between study groups.

**Fig. 3:**
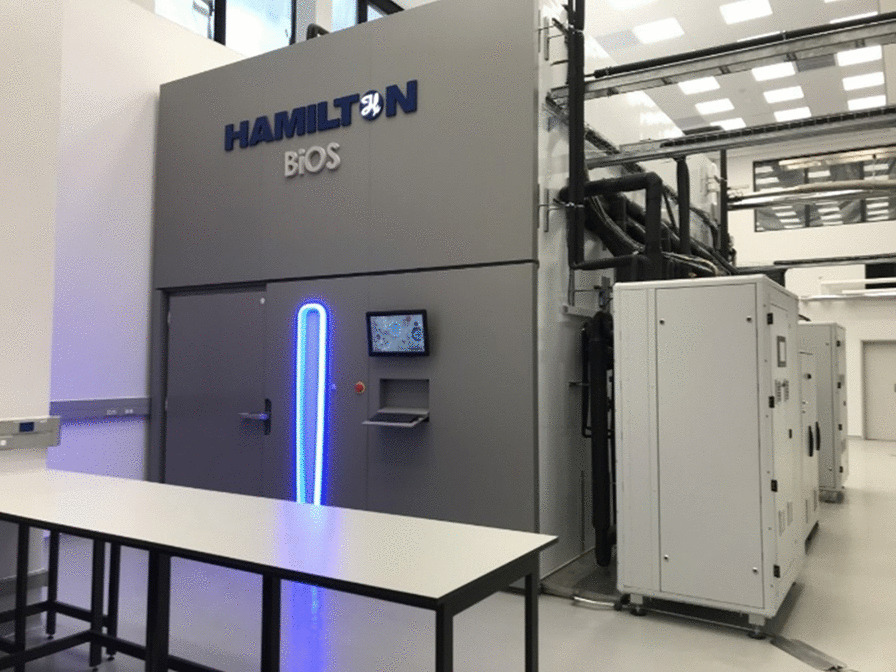
Hamilton BiOS at the Biorepository Unit, BMRI, Stellenbosch University

The remaining PBMCs will be stored at the BMRI Biorepository Unit (Fig. 3), [9] SU for future assays. Blood (2.5 ml) will be collected in PAXgene® RNA tubes and sent to SCRI for bulk RNA sequencing.

### Sputum collection (5 ml)

Sputum will be used to detect Mtb by GeneXpert MTB/RIF Ultra® assay, Auramine smear and liquid culture [Mycobacterial Growth Indicator Tubes (MGIT), BACTEC 960, Becton Dickinson] and will be collected by either spontaneous sputum production or sputum induction by hypertonic saline. Isolates from sputum cultures of all cases will be stored at − 80 °C for future analysis including strain typing.

### Bronchoscopy procedures

One of the unique features of the Cascade study is the collection of paired peripheral blood and BAL samples from comprehensively phenotyped study participants. This will allow the analysis of immune reactions at the site of infection (lung) in addition to the peripheral immune response in the blood. Bronchoscopies will be performed on all SARS Co-V-2 PCR negative active TB participants and all contacts and controls who do not test positive for SARS-CoV-2 by PCR at month 3 and who had QFT TB antigen minus nil 1 and 2 values either < 0.3 or > 0.5 IU/ml on both the screening and month 3 visits.

**Fig. 4:**
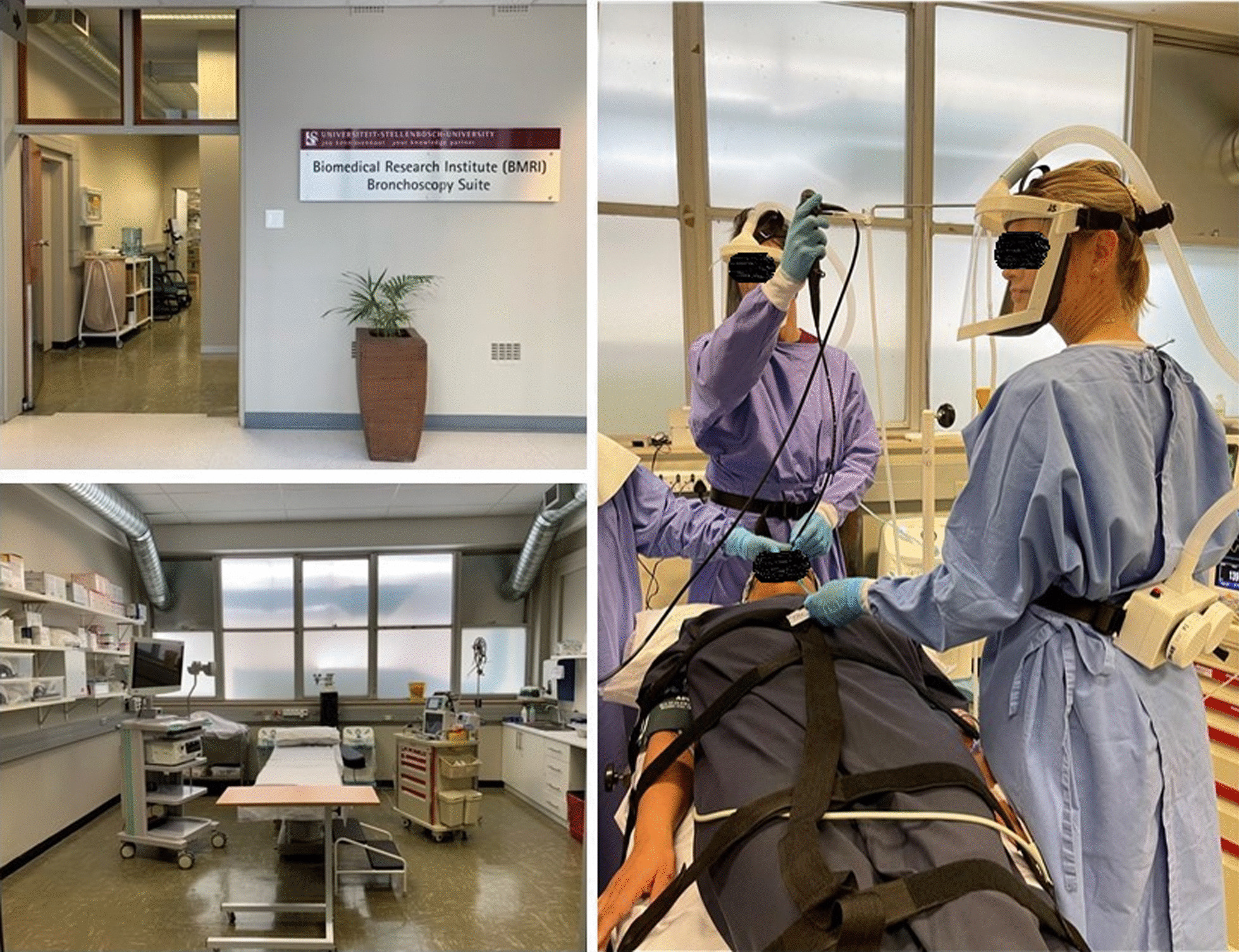
BMRI Research Bronchoscopy suite, Stellenbosch University

Bronchoscopies will be performed in the BMRI Bronchoscopy Suite [10], a research-dedicated bronchoscopy suite located at the Tygerberg Medical Campus (Fig. 4). BAL will be performed under procedural sedation by an experienced team of qualified pulmonologists, medical officers, and nursing staff. The 56 m^2^ suite is fully equipped with two EB-530TX video bronchoscopes (Fujifilm, Tokyo, Japan) with 3.2 mm working channels, an Eluxeo lite Processor (Fujifilm, Tokyo, Japan) with Multi Light Technology, a reprocessing unit (Soluscope, Augbane, France), and a drying cabinet (IDESO, Cape Town, South Africa). The suite is also equipped to monitor and sedate participants during bronchoscopy, with emergency equipment (including defibrillator), and an ultrasound unit (Sonosite, Fujifilm, Tokyo, Japan) with linear and curved transducers. The highest standards of infection prevention for personnel and participants have been implemented in the form of a ventilation system that ensures 12 air changes per hour, UV air sterilizers (Medicare Hospital Equipment, Cape Town, South Africa) and by using 3D-printed Powered Air Purifying Respirators (IDESO, Cape Town, South Africa) for all personnel. Strict procedures for reducing risk for SARS-CoV-2 transmission have been implemented, using the risk assessment and preventative measures detailed in Table 7. A 12-lead electrocardiogram (ECG) will be performed before the procedure on all participants with risk factors for cardiovascular disease.

**Table 7:** Transmission of SARS-Co-V-2 risks and relevant preventative measures

Setting	Individuals at risk	Nature of Risk	Transmission prevention plan
Transportation to study site by driver employed for the study and using study car	1. Driver 2. Uninfected participants using vehicle after infected participant	Risk of transfer of virus from infected participant’s bodily fluids, directly or via clothing, hands, bags etc. to vehicle surfaces, which may be transferred to next user Risk of droplet transmission in vehicle	1. All participants to clean hands with alcohol-based sanitiser prior to entry into vehicle 2. All participants to wear surgical mask during travel 3. Driver to always wear N95 mask and face-shield 4. Fitted transparent impermeable divider between driver and participant; participant to sit in the rear passenger seats of vehicle 5. Driver to clean door handles, seats, and other surfaces in vehicle as far as possible with alcohol-based disinfectant or 0.5% bleach solution directly after infected participant exits vehicle 6. No physical contact between driver and participant 7. Only one participant to be transported at a time, unless both participants are known to be SARS-CoV-2 positive and from the same household, or if they recently tested negative for SARS-Co-V-2 and have no symptoms 8. SARS-CoV-2 positive participants only to be transported in vehicles reserved for this purpose
Transfer from vehicle to waiting area	1. Uninfected participants 2. Passers-by, campus staff and students	Risk of transmission when infected participant encounters uninfected person, viral transmission on clothing, hands and droplets spread	1. Ensure dedicated ‘COVID-19 corridor for infected participants to move from vehicle, through dedicated entrance/exit to waiting area; out of dedicated entrance/exit from waiting area to vehicle; movement within trial site to be restricted to COVID-19-dedicated areas 2. All participants to wear surgical masks and practice social distancing, drivers escorting participants will ensure they do not stop *en route* to the waiting area; opportunities to interact with others minimised by use of the dedicated SARS-CoV-2 waiting areas, consultation rooms and corridors
Waiting area	1. Uninfected participants in waiting area at the same time as infected participants 2. Uninfected participants who sit in chairs or touch surfaces used by infected participants after they have left the area	Risk of transmission from infected participants to uninfected participants from chairs and other surfaces, and expectorated droplets	1. Separate waiting area reserved for infected participants, with minimal furnishings, all of which are amenable to washing with disinfectant 2. Four times daily disinfecting of surfaces in waiting area (including floors, walls, tables, and chairs) with alcohol-based disinfectant or 0.5% bleach solution 3. Participants to always wear surgical masks
Consultation rooms	1. Study nurses, nurse’s assistants, clinicians 2. Uninfected participants using consultation rooms after infected participants	Risk of droplet transmission Risk of transmission from surfaces and chairs	1. Dedicated consultation rooms for infected participants, uncluttered with only necessary stationery and equipment 2. Consultation rooms’ surfaces and chairs disinfected after each consultation with alcohol-based sanitiser or 0.5% bleach solution 3. Equipment (blood pressure cuff, thermometer etc.) wiped with alcohol-based sanitiser after each use 4. Study staff to wear surgical masks, face-shield or goggles, plastic aprons, and non-sterile gloves for the duration of the consultation. Gloves to be discarded and hands washed with alcohol-based disinfectant between participant encounters. Goggles and face-shields to be soaked in a bucket of disinfectant for 30 min at the end of a day. Aprons to be discarded on exiting the consultation room. Recommended donning and doffing procedures will be followed 5. Mobile perspex screens to be placed on desks in consultation rooms between participants and staff members
Bronchoscopy suite	1. Bronchoscopy clinical staff including endoscopist/pulmonologist, seditionist, bronchoscopy nurse, nursing assistant 2.Laboratory staff who collect specimens 3. Other participants waiting in recovery area	Risk of airborne transmission from particle aerosols during bronchoscopy Risk of transmission from surfaces Risk of droplet transmission from coughing post procedure	1. The bronchoscopy suite is equipped with an advanced ventilation system which provides a full air change every 5 min, fully extracting any aerosolised infectious particles 2. During the procedure every member of the clinical and laboratory team will wear the following PPE: a gown/single use laboratory coat (to be removed and discarded/washed after each procedure); a Powered Air Purifying Respirator (PAPR, which provides continuous N99-equivalent filtered air and full-face protection to the wearer); disposable gloves. If a PAPR is unavailable, then a combination of N95 and eye protection will be worn 3. Only the essential staff for the procedure will be allowed in the suite during the procedure and for 5 min afterwards 4. Doors between the suite and recovery room will be closed during the procedure and for 5 min afterwards to allow for a full air change in that area 5. The participant will don a surgical mask as soon as the bronchoscope is removed from their airway and continue to wear it throughout recovery 6. Participants who are waiting for their procedure will only enter the recovery area if it is empty of recovering participants (who may cough post-procedure) 7. All surfaces will be wiped/sprayed with sanitising solution after each procedure, and the whole suite will undergo daily wash with soap and water
Sample collection booth	1. Uninfected participants using the booth after infected participants 2. Staff who enter the booth	Risk of airborne transmission from particle aerosols Risk of transmission from surfaces	1. Dedicated sample collection booth for high-risk participants; separate collection booths to be used by uninfected participants 2. Sample booth to be fully disinfected twice daily, including walls, floors, and all surfaces, with alcohol-based disinfectant or 0.5% bleach solution 3. Staff members present for the sample collection process will wear N95 face masks, face shields or goggles, disposable gowns (with full sleeves) and non-sterile gloves. Masks, gloves, and gowns will be doffed and disposed after every use; shields and goggles will soak for 30 min in disinfectant solution before the next use. Recommended donning and doffing procedures will be followed
Participant bathrooms	1. Uninfected participants	Risk of transmission from aerosolised particles and from surfaces in the bathroom	1. Dedicated bathroom for SARS-Co-V 2 infected participants only 2. Surfaces to be cleaned and disinfected twice per day with alcohol-based disinfectant or 0.5% bleach solution 3. Adequate ventilation (> 15 air exchanged per hour) ensured in bathrooms
All participant and clinical areas	1. Cleaning staff	Risk of transmission from surfaces and via airborne droplets	1. Cleaning staff to wear a surgical mask, plastic apron, long rubber utility cleaning gloves (ideally up to elbow) that can be washed, goggles or face shield, closed work shoes 2. Cleaning staff to enter and clear participant areas only after 30 min or more have elapsed since a participant was in the area
Sample transport from consultation rooms, sample collection booth, to the laboratory	1. Drivers 2. Sample transporters	Risk of transmission from surface of sample container and bag	1. Sample runner and driver to wear non-sterile gloves which are discarded after single use, and clean hands with alcohol-based disinfectant after removing gloves 2. Samples to be double bagged after collection, and transported in sealed temperature-controlled container

BAL samples will be obtained from diseased areas of the lung in participants in the active PTB group as determined by chest radiograph, from any FDG-avid areas in contacts, and the right middle lobe in all other participants, unless this lobe is not accessible. Local anaesthesia using lignocaine gel in nostrils, spray in the throat and 2% solution to the vocal cords, trachea, and main bronchi (delivered through the bronchoscope working channel), not exceeding a total lignocaine dose of 4.5 mg/kg, will be used to provide additional analgesia [11]. Nasal or oral intubation will be used depending on the ease of access and participant’s comfort level. The airways will be inspected for any visible abnormalities before the bronchoscope is wedged in the target segment. A maximum of 240 ml of warmed sterile saline will be instilled in aliquots of 60 ml, and gently aspirated. Participants will be monitored for clinical deterioration, blood oxygen saturation, ECG changes, and non-invasive blood pressure (NIBP) before, during, and after the procedure until full recovery and will receive supplemental oxygen throughout the procedure. Light to moderate levels of sedation [level 1 or 2 on the University of Michigan Sedation Scale (UMSS); Table 8] will be attained using either midazolam or propofol combined with fentanyl intravenously.

**Table 8:** University of Michigan Sedation Scale

Score	Description
0	Awake and alert
1	Minimally sedated—patient drowsy and sleepy but rousable to verbal command
2	Moderately sedated—patient may be sleeping, but can easily be aroused by light tactile stimulation
3	Deeply sedated—only rousable by significant physical stimulation/repeated painful stimuli
4	Patient asleep—only rousable by significant physical stimulation/repeated painful stimuli
5	Not rousable—no response with significant physical stimulation

Before bronchoscopy, sterile saline will be passed through the bronchoscope for the subtraction of background DNA contamination during sequence analysis.

Bronchoscopy with BAL is usually considered safe when performed by experienced operators with appropriate training [12]. Complications can include bronchial irritation with bronchoconstriction (1–2%), pneumothorax (0.1–0.16%) [13] and minor hemoptysis (3%). The risk of severe bleeding is low (< 1%). The rate of complications with fatal consequences is < 1:5000, with only one reported case of death due to sepsis attributed to BAL [14]. The risk of bleeding and pneumothorax is lower in bronchoscopy with BAL compared to procedures such as transbronchial biopsy, which will not be performed in this study [15]. All potential risks associated with the procedure will be clearly explained to the participants during the informed consent process. After bronchoscopy, the participants will be monitored in the recovery room by trained personnel for at least 60 min, until fully awake, stable, and alert. In the case of an adverse event, this period will be extended until the participant is stable. In the case of a minor adverse event (i.e., nosebleeds or coughing) the participants will be allowed to return to their homes. Participants will be provided with a post sedation discharge sheet (Additional file 1). Should any complications arise, transport and treatment at the local hospital will be arranged. The participants will be given the contact number of the study nurse or clinician and will receive a follow-up phone call 72 h and 14 days post-procedure. In the case of severe adverse events, the participants will be admitted to the hospital until resolution.

BAL samples will be transported on ice to the SUN-IRG laboratory where processing will commence within 2 h of collection. End assays will include a range of single-cell and in vitro functional analyses, including single-cell RNA sequencing, Assay for Transposase-Assessable Chromatin (ATAC) sequencing, T-cell cloning, CyTOF phenotyping and 3-dimensional culture assays. BAL from SARS-CoV2 serology positive individuals will be assessed by way of similar end assays, to permit investigation into the effect of this viral respiratory infection on host immune susceptibility to Mtb and vice versa.

### PET-CT imaging

Month 3 PET-CT imaging of the contact and control arm participants will be performed at the Nuclear Medicine Research Institute Node for Infection Imaging (NII), SU, South Africa. The NII is a dedicated research PET-CT facility [16] hosted by SU within the Central Analytical Facilities and is situated at Tygerberg Academic Hospital. The unit is the only such facility in Africa accredited as a PET-CT Centre of Excellence by EANM Research Ltd (EARL) [17] and was designed specifically for research in infectious diseases such as TB. The NII is equipped with a Vereos PET-CT scanner (Philips, Amsterdam, The Netherlands) as well as a radio pharmacy for on-site synthesis and labelling of radiotracers. The NII is affiliated with the Division of Nuclear Medicine of SU which has more than a decade of academic PET-CT experience. The principle of ‘as low as reasonably achievable’ (ALARA) for radiation exposure will be followed. The dose of FDG is calculated at 2.8 mBq per kg, in the range of 185 mBq (5 mCi) to 257 mBq (7 mCi) and administered 60 min before imaging. The maximum total radiation from the proposed PET-CT scan is less than 1 Rem/10 mSv. This is lower than what is considered as ‘high’ annual radiation dosage and much lower than the maximal permissible annual research exposure of 5 Rem/year [18]) to 257 mBq (7 mCi) and administered 60 min before imaging. The maximum total radiation from the proposed PET-CT scan is less than 1 Rem/10 mSv. This is lower than what is considered as ‘high’ annual radiation dosage and much lower than the maximal permissible annual research exposure of 5 Rem/year [18].

A light meal will be provided after the PET-CT and bronchoscopy visits for all participants as these visits require participants to be fasting for more than 6 h before the procedure.

### Sample storage and management

Specimens collected will be stored in a fully automated, BMRI Biorepository Unit (Fig. 3) at SU [19]. This unit houses the BiOS Hamilton instrument [9], which has a storage capacity of 4–5 million samples based on tube sizes. Samples are stored at a constant temperature of − 80 °C, with a full audit trail and temperature log for each stored sample. The low energy footprint of the BiOS versus that of conventional freezers will result in a total energy saving of approximately 85%. The facility includes a dedicated laboratory for sample delivery and preparation. Automation will allow samples to be selected without temperature fluctuations that could degrade precious specimens. The system can read 1-D and 2-D barcodes, thereby improving sample tracking. It interfaces with information technology infrastructure and laboratory information management systems for complete automation of sample management. The facility is compliant with ISBER and ISO 20387:2018 and follows all ethical and regulatory requirements.

### Data collection and management

Participant information will in most cases be collected using standardised study questionnaires on password-protected tablet computers. Information will be captured directly into electronic case report forms on the REDCap platform [8], by study nurses and doctors trained on the study protocol. In some cases, such as documentation generated during the bronchoscopy, information will be captured on standardised paper study data collection forms, which will then be entered into electronic case report forms by a data-capturer.

Electronic case report forms will be programmed to provide warning messages and flags for violations of expected data types for dates, numeric data, and missing data entries. Manual data checks will be performed for essential data entries by a data quality control officer, according to the SU Cascade Household Contact study data management plan standard operating procedure (Additional file 2). The officer will log data queries for study nurses and clinicians to resolve, to ensure that missing or incorrect data are corrected in near real-time.

All participants will be assigned a study identification number and no identifying information will be available to study personnel who do not have direct contact with participants. Data will be stored in a secure, password-protected study database, on servers at the Faculty of Medicine and Health Sciences, SU. The REDCap data on the Hermes server is backed up daily to a secure server in a separate building and back-ups are archived at increasing intervals for 2 years.

A data dictionary was compiled (Additional file 3) to facilitate effective data sharing across various platforms and allow for the description of metadata and syntactic and semantic harmonization. All medical history or new medical findings or conditions diagnosed use ICD-10 (International Classification of Diseases, Tenth Revision) codes and these codes are captured in the eCFR (electronic Case Report Form). The Cascade Data Portal hosted by the UW will be the repository for processed data from this study. Of the 770 variables in the Cascade data dictionary, 232 will be shared with this data portal.

### Statistical analysis

Participants with missing data which prevents classification into one of the study outcomes groups will be excluded from the analysis. We will investigate the demographic characteristics of excluded participants for systematic biases.

Differences between study groups for continuous variables will be assessed with t-tests or generalised linear models for symmetrically distributed variables, and Wilcoxon rank sum tests for asymmetrically distributed variables. Differences between proportions will be assessed with Fisher’s exact tests. Complex relationships between continuous variables will be assessed with clustering techniques or network analysis methods. We will use correction methods for multiple comparisons when required.

For secondary analysis we intend to pool data from different study groups.

### Sample size considerations

Sample sizes were chosen to achieve sufficient power to address primary outcomes. Power was estimated using ANOVA power computation for a range of conditions with varying effect sizes, and varying numbers of groups, and samples per group, to account for variables not relevant to any specific group and for missing data due to measurement failure. Overall, there is greater than 80% power for effect sizes (Cohen’s f) that are in the medium to large range (0.2 < f < 0.4) for continuous-valued measures. Note that Cohen’s f is approximately ½ of Cohen’s d, therefore the power is approximately for any single group mean deviating from the mean of the other groups by at least one within-group standard deviation (SD).

Power to identify differences in RNAseq transcriptomic profiles across groups of size n = 20 exceeds 90% to detect the top 100 genes when fold-change exceeds 3. This calculation is based on conservative assumptions that the minimum average read counts among the 100 prognostic genes in the control group is 5, the maximum dispersion is 0.5, and the ratio of the geometric mean of normalization factors is 1; under these conditions, if the total number of genes tested is 10,000 then power exceeds 90% to detect these prognostic genes if their fold changes are at least 3, using two-sided 5%-level negative binomial exact tests at a false discovery rate (FDR) threshold of 20% [20].

## Discussion

The development of new and effective TB vaccines and host-directed therapies to prevent and treat TB disease is crippled by our limited understanding of the immune responses responsible for controlling and eradicating Mtb, and the lack of an effective way of confirming infection or vaccine-induced immunity.

Because cellular immune responses are critical for immunity to TB, and the relative contribution of humoral responses are less understood, the study of the cellular immune responses at the site of the disease, utilizing multidisciplinary approach to dissect protective cellular mechanisms is important. Through comprehensive immunologic phenotyping and intergroup comparison between individuals who are protected from TB infection in a high exposure environment, those who develop latent infection, those who develop subclinical disease (identified by PET-CT), active pulmonary TB controls and healthy unexposed controls, the Cascade household contact study will provide detailed cellular and transcriptomic datasets of host immune responses to Mtb infection. These data will advance efforts to develop reliable biomarkers for protective host responses to Mtb infection, and pave the way for faster, more reliable, and cost-effective testing of new vaccine candidates.

The Cascade Household Contact study takes place in a well-established TB research unit with structures in place for the recruitment, follow up and transport of participants from high incidence settings in the near vicinity. Combined with the availability of expertise and facilities in the SUN-IRG laboratory, the BMRI Bronchoscopy suite, the NuMeRI Node for Infection Imaging and the BMRI Biorepository Unit, a unique opportunity for the study of different phenotypes already exists. The high TB prevalence in the areas from which participants are recruited, and the support of local and provincial government clinics contribute to the suitability of the site to conduct the Cascade Household Contact study.

The recruitment of IGRA-negative individuals in the contact and control arms in a high incidence setting presents an anticipated challenge. Together with the additional exclusions at screening and month 3 due to IGRA TB-nil values within the ‘grey zone’, this leads to high screening numbers with high early exclusion rates. This not only leads to an increased burden of work but also higher costs. Arbitrary initial ‘grey zone’ values of 0.2–0.7 IU/ml were thus reviewed and adjusted to 0.3–0.5 IU/ml in Version 7.0 of the protocol.

Many studies recommend the use of Tuberculin skin tests (TST) to identify resisters within the population. Both PPD based Tuberculin skin tests (TST) and IGRA tests are indirect and imperfect ways to classify disease progression in Mtb infection in highly exposed adult populations [21, 22]. In regions like South Africa, where BCG vaccinations are routine, the specificity of the TST is approximately 60% as opposed to the much higher specificity (97%) in non-vaccinated populations [22, 23]. For this reason, TST will not be used in this study to identify resisters. IGRA is more useful in our settings as it is not influenced by BCG-vaccination status [22]. Due to IGRA tests having a high variability [23], an arbitrary ‘grey zone’ of TB-nil values 0.3–0.5 IU/ml will be implemented in this study and all values within this range would lead to exclusion of the participant from the study. Despite adding complexity to the recruitment process, the grey zone will increase the accuracy of phenotyping and make the results more robust. New developments in transcriptomics and the use of RNA analysis to identify highly predictive Mtb specific biomarkers can increase the accuracy of phenotyping the various stages of Mtb infection in future studies.

Pooling of data from a wide range of technologies used will provide a unique and multi-layered picture of the cellular immune response in TB. BAL samples will make the study of these responses at the first point of contact between the TB bacillus and the immune system possible and provide a panoramic view of the whole spectrum of disease in different phenotypes.

The Cascade Household Contact study is being conducted during the COVID-19 pandemic, a factor complicating the execution of the protocol on multiple levels. With the deferment of the National Strategic Plan to reach TB goals by 2022, TB programs were derailed by the diversion of resources to the COVID-19 response [1]. As a result, TB testing and diagnosis were dramatically reduced, and TB treatment delivery at healthcare facilities was far below the requisite standard to achieve an acceptable cure rate during the pandemic. Compounding the problem for research units is the periodic halting of recruitment, as lockdown restrictions are increased and eased in response to each new wave of COVID-19.

Since the effect of previous SARS-CoV-2 infection on immune responses to TB is unknown, the SARS-CoV-2 cohort groups will be studied separately. This will provide insight into the dynamics between recent SARS-CoV-2 infection and immune mechanisms against TB.

No Mtb strain typing is done in this study and different immune mechanisms for different TB strains will thus not be studied.

By the in-depth study of site-specific and systemic immune responses to Mtb exposure in different individuals, the Cascade IMPAc-TB Household Contact Study will contribute to the scientific understanding of the successful human immune response to Mtb infection, paving the way for utilisation of this knowledge to increase innate and vaccine-induced immunity in high incidence settings. Combined with data from the other studies conducted by collaborating experts on NHPs and mice, a more comprehensive understanding will be possible, facilitating the development of effective TB vaccines and prevention strategies.

## Supplementary Information

**Additional file 1.** Cascade bronchoscopy source document and discharge summary.

**Additional file 2.** Cascade data management plan.

**Additional file 3.** Cascade data dictionary.

## Data Availability

Since this is a protocol, data are not available yet but will be made freely available for non-commercial use on ImmPort website [24], NIAID-supported database after completion of data collection and analysis. Any unused biological samples will be available for non-commercial use and maybe requested from Dr. Walzl (gwalzl@sun.ac.za), the Principal Investigator of the study at Stellenbosch University, who will then forward the request to Dr. Urdahl, the Principal Investigator of the main study, and the Contracting Officer at NIAID, for final approval.

## Abbreviations

ALARA: As low as reasonably achievable
ATACSeq: Assay for Transposase-Accessible Chromatin using sequencing
BAL: Broncho-alveolar lavage
BMGF: Bill and Melinda Gates Foundation
CAB: Community Advisory Board
COVID-19: Coronavirus disease 2019
CRF: Case report form
CRW: Community Research Worker
CyTOF: Cytometry-by-time-of-flight
ECG: Electrocardiogram
EDCTP: European and Developing Countries Clinical Trials Partnership
eCRF: Electronic case report form
FDG: Fluorodeoxyglucose
FMHS: Faculty of Medicine and Health Sciences
Hb: Haemoglobin
HbA1c: Haemoglobin A1c
HCC: Healthy community control
HHC: Household contact
HIV: Human Immunodeficiency Virus
HREC: Health Research Ethics Committee
ICD 10: International Classification of Diseases, Tenth Revision
ICF: Informed Consent Form
IgG: Immunoglobulin G
IGRA: Interferon Gamma Release Assay
IMPAc-TB: Immune Mechanisms of Protection Against *Mycobacterium tuberculosis* Centre
MGIT: Mycobacteria Growth Indicator Tube
Mtb: *Mycobacterium* *tuberculosis*
NaHep: Sodium Heparin
neg: Negative
NHLS: National Health Laboratory Service
NHP: Non-human primate
NIAID: National Institute of Allergy and Infectious Diseases
NIBP: Non-invasive blood pressure
NIH: National Institutes of Health
NII: NuMeRI Node for Infection Imaging
OHSU: Oregon Health and Science University
PBMC: Peripheral Blood Monocyte Cells
PET-CT: Positron emission tomography/computed tomography
PCR: Polymerase chain reaction
POC: Point of Care
pos: Positive
PPD: Purified Protein Derivative
PTB: Pulmonary tuberculosis
REDCap: Research Electronic Data Capture
RNAseq: Ribonucleic acid sequencing
SA-DSI: South African Department of Science and Innovation
SAE: Serious Adverse Event
SAMRC: South African Medical Research Council
SANRF: South African National Research Foundation
SARS-CoV-2: Severe Acute Respiratory Syndrome coronavirus 2
SATBBI: South African Tuberculosis Bioinformatics Initiative
SCRI: Seattle Children’s Research Institute
SOP: Standard operating procedure
βHCG: Beta Human Chorionic Gonadotropin
SU: Stellenbosch University
SUN-IRG: Stellenbosch University Immunology Research Group
TB: Tuberculosis
TST: Tuberculin skin test
UMSS: University of Michigan Sedation Scale
USNRC: United States Nuclear Regulatory Commission
UW: University of Washington
