# Supplementary material for: Cascade Immune Mechanisms of Protection against Mycobacterium tuberculosis (IMPAc-TB): study protocol for the Household Contact Study in the Western Cape, South Africa
Source: BMC Infect Dis. 2022 Apr 15;22:381. doi: 10.1186/s12879-022-07349-8 (PMC9012070; doi:10.1186/s12879-022-07349-8)
Supplement: Supplementary file 1 — Additional file 1. Cascade bronchoscopy source document and discharge summary. [file 12879_2022_7349_MOESM1_ESM.pdf]

CASCADE BRONCHOSCOPY SOURCE DOCUMENT:

PID: ______________________________

Confirmed SARS-CoV-2 negative on PCR within last 7 days? : Yes ☐ No ☐

1. Medical history:

Any changes in Medical history/ Medication since screening visit? Yes ☐ No ☐
If Yes: Details:
Current Medication:
Cardiovascular Disease risk Factors? Yes ☐ No ☐ If Yes, Specify and do ECG
Smoking history:
Drug use history:
Surgical History:
Prior Adverse reaction to local or general anaesthetic: (own or familial)
Allergies:

Special investigation	Done	Date	Comment:	Preclude safe procedure?
SARS CoV-2 Serology	☐Yes ☐No	DD/MMM/20YY		☐Yes ☐No
CXR	☐Yes ☐No	DD/MMM/20YY		☐Yes ☐No
ECG	☐Yes ☐No	DD/MMM/20YY	Indication:	☐Yes ☐No
Safety blood tests	☐Yes ☐No	DD/MMM/20YY		☐Yes ☐No
PET/CT	☐Yes ☐No	DD/MMM/20YY		☐Yes ☐No
Other	☐Yes ☐No	DD/MMM/20YY		☐Yes ☐No

(Tick)	**Karnofsky score** (if <60, do not perform bronchoscopy)
	**100%** - normal, no complaints, no signs of disease.
	**90%** - capable of normal activity, few symptoms or signs of disease.
	**80%** - normal activity with some difficulty, some symptoms or signs.
	**70%** - caring for self, not capable of normal activity or work.
	**60%** - requiring some help, can take care of most personal requirements.
	**50%** - requires help often, requires frequent medical care.
	**40%** - disabled, requires special care and help. 30% - severely disabled, hospital admission indicated but no risk of death.
	**20%** - very ill, urgently requiring admission and supportive measures of treatment.
	**10%** - moribund, rapidly progressive fatal disease.

2. Examination:

**Baseline vitals:**	
BP: ☐☐☐/☐☐☐<br>Pulse: ☐☐☐ /min	Saturation (room air): ☐☐☐%<br>Resp Rate: ☐☐/min
Weight: ☐☐☐,☐ kg	

**Targeted physical examination:**	
Pertinent signs of disease	☐None<br>☐Clinically wasted<br>☐Pallor<br>☐Clubbing<br>☐ Palpable lymphadenopathy<br>☐Other If other specify:
Positive findings on auscultation:	☐Yes ☐No<br>If yes, specify:
Other Resp findings	☐Yes ☐No<br>If yes, specify**:**
Cardiovascular	☐Normal ☐Abnormal<br>If Abnormal, specify**:**
Mouth Opening	

Teeth	
Mallampati (circle)	1 2 3 4
Neck – range of motion	
ASA rating: (circle)	I II III IV E

Print name: _____________________Signature: _____________________ Date: DD/MMM/20YY

Designation (circle correct) Study staff /TBH staff

3. Bronchoscopy:

Consent signed in ICF,<br>Understanding confirmed	☐Yes ☐No N/A ☐
Fasting >6hours solids and opaque fluids; >4 hours clear fluids	☐Yes ☐No

**Inhalation**: (circle) O_2 Flow Rate: ____l/min Air Other	
**Fluid :**	
Site of IV line:	Type of IV Fluid:
Total volume fluids given: ______________ml	IV cannula size: (circle) : 24G 22G 20G 18G
**Monitors:** (circle) ECG Pulse oximeter NIBP Other	
**Ventilation:** (circle) Spontaneous Manual	

**Time**													
220													
200													
180													
170													
160													
150													
140													
130													
120													
110													
100													
90													
80													
70													
60													
50													
40													
30													
20													
10													
SpO2													
FiO2													
Sedation Level													

**<u>University of Michigan Sedation Scale</u>**	
0	Awake and Alert
1	Minimally sedated – Patient drowsy and sleepy but rousable to verbal command
2	Moderately sedated – Patient may be sleeping, but can easily be aroused by light tactile stimulation
3	Deeply sedated – only rousable by significant physical stimulation/ repeated painful stimuli
4	Patient asleep – only rousable by significant physical stimulation/repeated painful stimuli
5	Not rousable – no response with significant physical stimulation

Was bronchoscopy performed?	☐Yes ☐No If no, specify:
Date of bronchoscopy:	DD/MMM/20YY
Bronchoscopy nurse:	
Bronchoscopist:	
Sedationist:	
Bronchoscopy Theatre: (Circle)	BMRI A5:TBH

Start time of sedation: ___:___	Start time of procedure: ___:___
Duration of procedure (min)	
Access route	☐Right nostril ☐Left Nostril ☐Mouth<br>Comment:
Local Lignocaine	☐Yes ☐No
Lignocaine gel in Nostrils	☐Yes ☐No Right: ☐ Left: ☐
Lignocaine spray in throat (mg)	
Lignocaine cords/trachea (mg)	
Lignocaine total (mg)	

*Remicaine gel 2% (2g/100g), 1 gram of gel is 20mg of lignocaine, ** Xylocaine 10mg/metered dose (or 0.1ml) delivers 10mg lignocaine per spray. ***Each dose of lignocaine delivered through the bronchoscope working channel is 20mg (2ml of 1% lignocaine, 10mg/ml) Maximum safe dose = 4.5mg/kg (or 300mg for a 65kg adult)

Systemic sedative administered? ☐Yes ☐No			Dosage for weight =________kg	
Drug:	Total Dose:	Route	Recommended for bolus	
Propofol ☐Yes ☐No			0,5 mg/kg<br>over 3-5 minutes<br>= _________mg	
Fentanyl ☐Yes ☐No			0,25 µg/kg<br>=__________ µg	2 µg/kg<br>=__________ µg
Midazolam ☐Yes ☐No			0.05-0.1 mg/kg<br>[max 2 mg]<br>= __________mg	3mg
Any other medication? ☐Yes ☐No<br>Details:				

Bronchoscopy findings:

	Observations (circle)	Comment if abnormal
Cords	Normal/Abnormal	
Mucosa	Normal/Abnormal	
Secretions	None/some/copious<br>Clear/opaque/purulent/bloody	Arising from specific segment?
Bronchial Anatomy	Normal/variant of normal/abnormal	
BAL fluid	Clear/bloody/milky/black	
Other important findings		

Lung Lobe used	☐RUL ☐RLL ☐RML<br>☐LUL ☐LLL ☐ Lingula
Complications	☐ Yes ☐ No<br>If yes, was it serious? ☐ Yes ☐ No<br>Comment:
Volume in (ml)	
BAL fluid out (ml)	
Other procedures (eg bronchial brush/ Biopsy?)	☐Yes ☐No<br>Comment:
Ultrasound performed after procedure?	☐Yes ☐No<br>Comment:
BAL sample (±150 ml) transported to lab **on ice**<br>Time in cooler box: ___:___	☐Yes ☐No<br>Comment:

Print name Signature DD/MMM/20YY Designation

POST-PROCEDURAL ASSESSMENT:

	Yes	No	Comment
Are the blood pressure and heart rate stable/within pre-bronchoscopy range?			
Can the patient swallow and cough?			
Can the patient walk without feeling dizzy or faint?			
No nausea or vomiting.			
Is the patient breathing comfortably and of normal color?			
Is the patient awake and behaving appropriately?			
No active bleeding from nose or mouth.			
Have verbal postoperative instructions been given and explained to both the patient and caretaker?			
Is the patient pain free?			
Have possible complications been explained?			
Will the patient be transported home by study staff?			
TB Positive participant referred to CHC and provided with referral letter and copies of reports?			
Any other complaints?			
Copy of discharge summary given to participant?			

Signature: ____

Date: DD/MMM/20YY Time: _________ Designation: Study Clinician

UNIVERSITEIT STELLENBOSCH UNIVERSITY

Division Molecular Biology and Human Genetics
Clinical Standard Operating Procedures

Post Bronchoscopy Discharge Summary

(Please supply participant with a copy to take home)

The below participant underwent a bronchoscopy with broncho-alveolar lavage for research purposes at the University of Stellenbosch, Bio-medical Research Unit

Referral site: ___________________________ **Study name:** ________________________________

PID: ___________________________________ **Date of procedure:** dd / mmm / 20yy

Lung lobe(s) used for lavage*: ______________ **Other procedures performed:** ________________

**Residual fluid in this part of the lung will be visible on chest radiograph for up to 24 hours after the procedure*

Sedation used during procedure: Propofol / Midazolam / Fentanyl / Other ____________________

Lignocaine spray used: ____

Vital signs on discharge: HR: ______/min RR: ______/min BP: ____/____ Sats: _________%

Any complications: ____

____

Any abnormalities noted: ____

Possible minor adverse events from this procedure include discomfort of the nose and throat,

coughing, low grade fever, minor epistaxis, chest pain or speckled haemoptysis.

Please contact the number below if symptoms are severe, the patient has dyspnoea, or symptoms

persist for > 24 hours.

In case of emergency contact:

Name: ________________________________ Contact number: ______________________________

Facility: _______________________________

Assessed by: ___________________________ Signature: ___________________________________

Date: dd / mmm / 20yy Time: ____h____
