# Supplementary material for: Cascade Immune Mechanisms of Protection against Mycobacterium tuberculosis (IMPAc-TB): study protocol for the Household Contact Study in the Western Cape, South Africa
Source: BMC Infect Dis. 2022 Apr 15;22:381. doi: 10.1186/s12879-022-07349-8 (PMC9012070; doi:10.1186/s12879-022-07349-8)
Supplement: Supplementary file 2 — Additional file 2. Cascade data management plan. [file 12879_2022_7349_MOESM2_ESM.pdf]

SU IMMUNOLOGY RESEARCH GROUP

Division Molecular Biology and Human Genetics

<table>
<tr><td colspan="4">DATA MANAGEMENT PLAN: CASCADE STUDY</td></tr>
<tr><td>SOP#:</td><td>Version #: 1.0
(Supersedes version 0.0)</td><td>Effective Date:
(This document will be reviewed in two years from effective date)</td><td></td></tr>
</table>

Author:	Reviewers: *(Signature confirms that the reviewers agree with the content of the document)*	Approved by Principal Investigator: *(Signature confirms final approval)*
(Signature & Date)<br><br>Kim Stanley<br>Data Manager<br>SU-IRG	(Signature & Date)<br><br>Prof Gian van der Spuy<br>Lead Data Manager<br>SU-IRG	(Signature & Date)<br><br>Prof Gerhard Walzl<br>Principal Investigator<br>SU-IRG<br><br>**Approved by QA:**<br>*(Signature confirms document ready for distribution and training)*<br><br>(Signature & Date)<br><br>Liesel Muller<br>Quality Assurance Officer<br>SU-IRG

TABLE OF CONTENTS

1. SCOPE 3
2. RESPONSIBILITIES 3
3. RISK ASSESSMENT 3
4. SUMMARY: WORK FLOW 3
5. PROCEDURE 3
5.1. INTRODUCTION 3
5.2. DATA CAPTURE AND ENTRY 4
5.3. DATABASE TESTING AND VALIDATION 5
5.4. DATABASE USER RIGHTS 5
5.4.1. Forgotten Password Management 5
5.5. DATA REPORTING AND EXPORT 6
5.5.1 Export of de-identified data 6
5.6. DATA VALIDATION 7
5.6.1. DATA DISCREPANCIES - MANUAL MISSING DATA 7
5.6.2. DATA DISCREPANCIES – DATA QUALITY RULES 12
5.6.3. RESULTS DATA VERIFICATION 13
5.6.4. RESULTS DATA MISSING 14
5.7. DATA DICTIONARY AND CODEBOOK 14
5.7.1 Database Changes & Data Integrity 15
5.8. DATA TRACK CHANGES 16
5.9. LOGGING AND AUDIT TRAIL 16
5.10. SECURITY 17
5.11. DATA ARCHIVAL 19
5.12. DATA STORAGE & BACK-UP 20
6. DEFINITIONS 20
7. REFERENCES 20
8. SUPPORTING DOCUMENTS 20
9. AVAILABILITY 21
10. DOCUMENT HISTORY 21

1. SCOPE

This SOP covers all the elements of database and data management for the CASCADE study. This includes data entry, validation and verification, security, storage and back-up, coding/dictionary, logging trails and archival.

2. RESPONSIBILITIES

The various aspects of the study's data management, including those mentioned in the scope, will be the responsibility of the field workers/study nurses, Study Clinician, Data Managers (DM) and lab personnel.

3. RISK ASSESSMENT

Not applicable.

4. SUMMARY: WORK FLOW

Not applicable.

5. PROCEDURE

5.1. INTRODUCTION

Volunteers for the CASCADE Study are recruited and followed-up from multiple clinics in as well as community health centres/day hospitals in Cape Town, South Africa. The study data for this human cohort of the Cascade IMPAc-TB Center consortium is managed at the University of Stellenbosch, SUN Immunology Group. This is the base for all those responsible, including the study nurses and clinicians, laboratory staff, managers, P.I., senior scientists and control officers. The University of Stellenbosch data analysis and database team will work closely with the consortium's Data and Resource Management System (DRMS). The study duration is seven years.

5.2. DATA CAPTURE AND ENTRY

Data for the study at this center is captured directly into a web based data management system known as REDCap (Research Electronic Data Capture) by the study fieldworkers and clinician (clinical data) and the laboratory stuff (lab and results data). Each study fieldworker has a tablet which uses an Android mobile operating system. Using 3/4G data sim cards and a browser they can connect to the web and REDCAP site hosted on the division's server. An added plug-in, called Project Laboratory Information Management System (PLIMS) was developed by the Lead Data Manager and is used to manage the subject barcode generation, follow-up schedules, and sample and storage management.

The scheduling module for longitudinal studies is enabled for CASCADE, as volunteers are followed-up over several events. The study also has multiple arms; ARM1: Screening, ARM2: TB Cases, ARM3: Comm. Controls, ARM4: Household Contacts and ARM5: HHC Subclin. The subjects in the different arms have different requirements (schedules and/or different forms) and this is facilitated by arm assignment. The arms/events are downloaded and saved as a .csv (comma separated values format) file. If there is a change to events or arms, an updated version should be saved.

Aside from the directly entered clinical data, there will be data taken and transcribed from external sources (e.g. laboratory reports, participant clinic records). This will be managed differently from the directly entered clinical report data. All such source documents will be stored either electronically in a centralized folder or printed out and kept in study files.

The Data Manager will program and customize the data entry screens for each form used in the study. CASCADE report forms are divided into separate instruments in REDCap. Download and save the instrument mappings as a .csv file. The mappings show the forms designated for each arm and event. If there is a change to this mapping, download and save the updated version.

5.3. DATABASE TESTING AND VALIDATION

Test data must be entered into the database in order to thoroughly check that the most optimal and appropriate field types were used as part of the design, the selected text field validations are correct, and that any branching logic is functioning correctly. This test data will be deleted when the study goes into production and true data is entered. A Database Validation Report will be written detailing the process and provides documented proof that the database performs the functions required.

5.4. DATABASE USER RIGHTS

Only users with a valid REDCap account can be granted access to the study. The Data Manager will use the User Rights page to grant users access to this study and for managing the user privileges of those users. The DM will create roles to which they will assign users. The roles groups together and categorizes users with the same privileges. This study database has five main roles; Admin, Clinicians/coordinators, FieldNurse, LabTech and Scientist. The Admin have highest level privileges, including Project Design and Setup, User Rights, Data Access Groups and full export rights. The LabTech users can only view and edit the relevant laboratory/results forms.

5.4.1. Forgotten Password Management

Users should notify REDCap admin of forgotten passwords via email. Admin can click on Control Center > Browse users and enter the username. Under user information > Statistics & Other information > reset user’s password. Click on the reset button. This will prompt an email to the username with a link to reset their password.

5.5. DATA REPORTING AND EXPORT

Reports of all or parts of the study data can be created and customized using the "**Reports**" application. Requests for data from REDCap can be made to the Data Manager or users with report rights can create new reports. This is achieved by clicking on "**Data Exports, Reports, and Stats**" application and hit the "**Create New Report**" tab. Then take the following actions:

1. Provide a proper name for the report.
2. Set the View and/or Edit User Access.
3. Select which fields or entire instruments you want to include in your report.

4. Set up optional filters and/or additional filters to select the appropriate records.
5. Set up an optional order for your report.
6. Hit "Save Report"

The data can then be exported in the following formats:
CSV/Microsoft Excel (raw)
CSV/Microsoft Excel (labels)
SPSS Statistical Software
SAS Statistical Software
R Statistical Software
STATA Statistical Software

Unless otherwise requested, the labels option should be selected for exports and analysis. Report user rights should mirror that of instrument access rights.

5.5.1 Export of de-identified data

There are a number of different options available for ensuring de-identification of sensitive data:

a) Tagging known identifiers in the Data Dictionary with the option to then remove all on export.
b) Hash the Record ID field. This converts the record name to an unrecognizable value.
c) Remove un-validated text fields i.e. text fields other than dates, numbers etc.
d) Remove notes/essay box fields
e) Remove all date and date/time fields OR
f) Shift all dates by value between 0 and 364 (shifted amounts depends on algorithm for each record). Date shifting leaves the project record intact and will not affect the actual saved dates in the project. It merely alters the dates in their resulting format when performing a data export in REDCap (if the date shifting option is enabled). The shifted dates will always be the same for each record when performing a data export. The dates are shifted back in time up to 364 days, in which the shifted value is based on the Record ID using a complex algorithm. Because the shifted value is based on the record name, the amount that dates are shifted is unique for each record, but the shifted amount is the same within each record to maintain internal consistency.

Date shifting is important for de-identification because dates, like name and social security number, are identifiers that can be used for identifying an individual and thus possibly exposing confidential personal information. Date shifting prevents any dates from being used as identifiers for each project record while preserving the interval between dates.

5.6. DATA VALIDATION

5.6.1. DATA DISCREPANCIES - MANUAL MISSING DATA

At the end of each instrument is a Form Status - complete field. The study nurses are required to select one of the following options:

Table 1.

**Status**	**Radio Dial Colour**	**When Selected**
Incomplete	Red	When any field in an instrument is missing or pending.
Unverified	Yellow	When any result field in an instrument is entered but not verified OR clinician has reviewed fields
Complete	Green	When ALL fields in an instrument are entered.

Once a week, the Data Manager will run the "**DQ: Study IDs & dates for Screening**" report and the "**DQ: Study IDs, arm & event name & dates for follow-up events"** report. These reports are sorted by date, oldest to newest so it's easy to establish which IDs and events where completed in that week. Using this list, a visual inspection will be done of all instruments and fields to confirm their form status. If a form is marked as 'complete' but has missing or pending fields, the QCO or DM will change this to an 'incomplete' status. See Table 2 for a list of instruments that will have incomplete status until filled with results data. These need only be checked that they have not been given a complete status erroneously.

Table 2.

**Instrument Name****
• POC tests
• CXR
• Sputum GeneXpert Ultra Result
• QFN-Plus Test Results
• Sputum Smear Test Result
• Sputum TB Culture Result
• HIV Testing (if clinic results not available)
• PET/CT Result
• GeneChecker SARS-CoV-2 Rapid PCR Results [NHLS]
• SARS-CoV-2 Serology Results [NHLS]

Once a week, the Data Manager will check for any missing fields by going through that week's complete instruments, as indicated by the green status colour. When finding such a discrepancy, the DM will open and initiate a query using the Data Resolution Workflows (DRW) option. To do this click on the balloon icon to the left of the specific field. This opens a workflow window (see Figure 1.). The workflow starts with the date and time of the user that first created the entry. The data is verified by default but if it is for example missing, click on the "open query" radio dial. Assign the query to the field worker who was responsible for the data entry. Add an appropriate comment such as "data missing" and save.

It is the responsibility of the study field worker/data clerk/clinician to regularly check the "Data Resolution Dashboard" found by clicking on the "Resolve Issues" application (see Figure 2.). Using the Filters, select the Open/Unresolved issues and their user name.

This dashboard will indicate for which record a query was opened, which field, by whom, any comments and a hyperlink to the record and DRW window. The specific user will need to address the query, for example by filling in the missing data field, then update by selecting a response option and/or comment. The Data Manager will perform the last step in the workflow, which is to close the query once satisfied it is resolved. Closed/Resolved queries will be saved together and accessed via a filter search. The days a query has been open is also stated in the dashboard and must not exceed 30 days unless unusual circumstances are preventing data to be entered.

Figure 1.

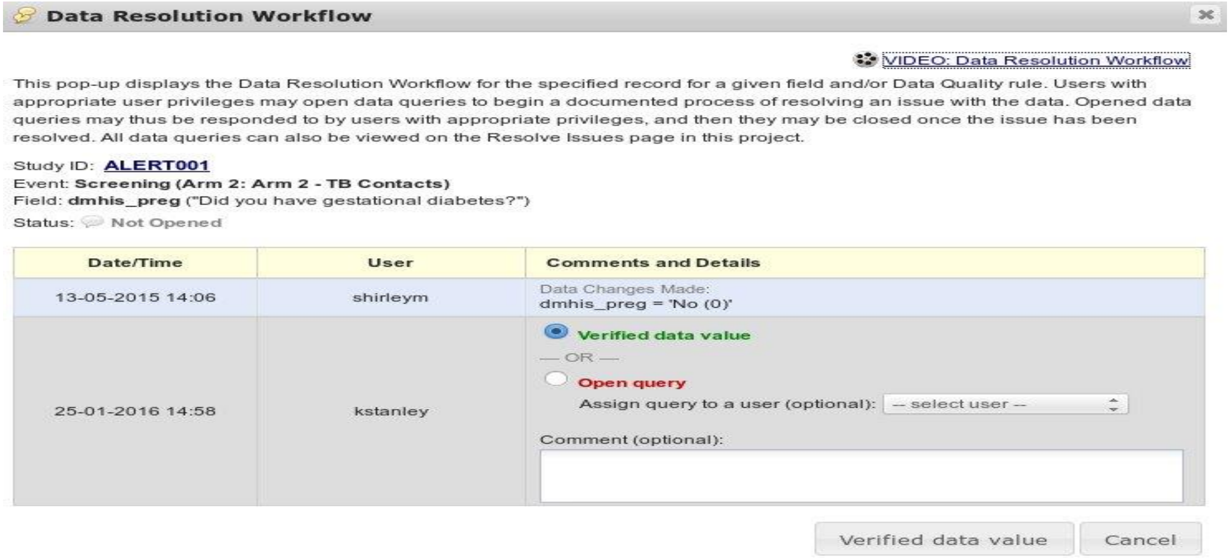

Figure 2.

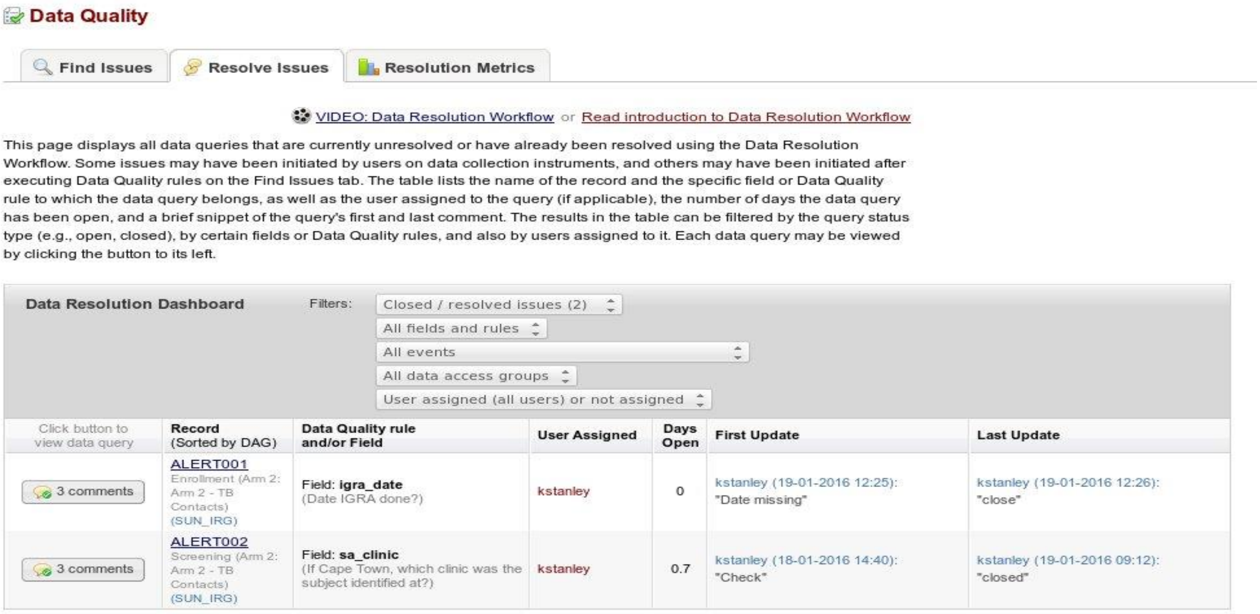

At the end of each month the Data Manager will download the dashboard of resolved issues and save.

Once monthly the Data Manager will use the "Resolution Matrix" function which summarizes the statistics and metrics for all the queries. The data from this matrix will be used to fill in a Data Resolution Workflow Summary log (see point 8. Supporting Documents).

Once monthly take and save a screenshot of the "Top 5 Queried Fields". These "most often queried" fields need to be communicated to the study nurses and clinician if a trend is seen. This will help to identify any field(s) that are being incorrectly interpreted by nurses and/or participants. It can also help identify fields which are no longer relevant.

Any other manual discrepancies, other than missing data that are discovered, are to be resolved using the same DRW function or if not possible directly through REDCap, logged with a FIT-001 Data Problem Summary form (see point 8. Supporting Documents).

Once a month the Record Status Dashboard will be checked. This is a table listing all existing records/responses and their status for every data collection instrument (and if longitudinal, for every event). This is an easy way to check the current status of all forms. Any incomplete forms/fields that have remained so for extended periods should be queried by initiating a DRW.

5.6.2. DATA DISCREPANCIES – DATA QUALITY RULES

The Data Manager is required to run the "Data Quality Rules" C, D, F - I found in Applications every week (see Figure 3.). These are pre-defined rules which find discrepancies when you click on the execute button. Take a screenshot of all rules, once executed, with data and time and save. Fill out a Data Quality Rules Discrepancy Log if there are any discrepancies (see point 8. Supporting Documents). The Data Manager must attempt to resolve the displayed discrepancy and/or open a query via the DRW window which will alert the responsible study field worker/data clerk of the issue. Additional Data Quality rules can be created and run in a similar manner to the provided built-in rules.

Figure 3.

Data Quality Rules

Execute rules: All | All except A&B | Clear

Apply to: -- All records --

	Rule #	Rule Name	Rule Logic (Show discrepancy only if...)	Real-time execution ?	Total Discrepancies
	A	Blank values*	-		Execute
	B	Blank values* (required fields only)	-		Execute
	C	Field validation errors (incorrect data type)	-		Execute
	D	Field validation errors (out of range)	-		Execute
	E	Outliers for numerical fields (numbers, integers, sliders, calc fields)**	-		Execute
	F	Hidden fields that contain values***	-		Execute
	G	Multiple choice fields with invalid values	-		Execute
	H	Incorrect values for calculated fields	-		Execute
	I	Fields containing "missing data codes"	-		Execute

5.6.3. RESULTS DATA VERIFICATION

Instruments and/or individual fields that are entered from external source documents are to be assigned an unverified or yellow status. The DM will run a number of individual reports (indicated by a DV prefix) once monthly, pulling up these selected fields together with their status to identify which need verification. A complete form status can only be selected once the entered data is checked to match exactly against the source documents. If the value entered does not match, open a DRW and direct query to the lab staff responsible. They will need to re-enter the correct value within 30 days and the value is then re-verified. Please reference Table 3 below for list of instruments/fields requiring verification for CASCADE. Log that verification was done, monthly, using a Results Data Verification Log (see point 8. Supporting Documents).

Table 3.

**Instrument**	**Field (s)**
POC tests	HbA1c Result *if done*
HIV Testing	HIV ELISA test result *if done*
QFN Plus Test Results	ALL
Sputum Smear Test Result	ALL
Sputum TB Culture Result	ALL
Sputum GeneXpert Ultra Result	ALL
GeneChecker SARS-CoV-2 Rapid PCR Results [NHLS]	ALL
SARS-CoV-2 Serology Results [NHLS]	ALL

5.6.4. RESULTS DATA MISSING

Once monthly the same data verification reports are run but filtered on ‘incomplete’ status rather than ‘unverified’. This will show results that are missing. Depending on the result type, the time interval from sample collection needs to be taken into consideration before initiating a DRW to the relevant user. For example, sputum culture result’s form status can potentially only be changed to unverified after a period of 40+ days. Record these missing results in a spreadsheet, noting the action taken and date of resolution. Log that missing data was identified, monthly, using a Missing Results Data Log (see point 8. Supporting Documents).

5.7. DATA DICTIONARY AND CODEBOOK

The Codebook is a human-readable, read-only version of the project's Data Dictionary and serves as a quick reference for viewing the attributes of any given field in the project without having to download and interpret the Data Dictionary. Note: Checkbox fields have their coded values displayed both in the format defined by users in the Online

Designer/Data Dictionary as well as in the extended format seen in data imports and exports (i.e. field_code).

The Data Dictionary is a specifically formatted spreadsheet (.csv format) containing the metadata used to construct data collection instruments and fields. Save a downloaded copy of the Data Dictionary with the date in the file name. The revision history is available as each altered data dictionary is available for download from Project Setup < Project Revision History.

5.7.1 Database Changes & Data Integrity

If any changes are made to the design of the study in REDCap after going into production, the changes need to be tested as described previously for appropriateness and functionality by a Data Manager. A Data Dictionary/Codebook Change Log documenting the changes should be filled in by the Data Manager and saved (see point 8. Supporting Documents). The log should be uploaded to the share file system and the link sent via email to the relevant staff.

Before any major changes to the database are made by the data manager, a full data export using the Data Export Tool should be done. This is as a precaution against potential data loss. All changes are made in draft mode. After they are submitted, the changes are reviewed and depending on the type of change, automatically approved. REDCap will however ask for further review and confirmation via the Project Modification Module if they could potentially result in critical issues (e.g. deleting fields, re-coding multiple choice field options, label mismatch etc.). In this module the critical issues are detailed i.e. how many fields/forms to be added or deleted and how many records are affected. If everything looks correct, and you wish to go ahead with the changes, click the Commit Changes at the bottom of the page. To reject the changes made, click the Reject Changes button, which will place the project back in Draft Mode for the users to continue modifying or correcting any errors. To erase all

the drafted changes made and revert back to before Draft Mode was enabled, select Remove All Drafted Changes.

These changes made in Draft Mode will result in a change in the Data Dictionary. The latest version should then be downloaded and saved.

5.8. DATA TRACK CHANGES

Every entry for all variables/fields in the database has a tracked history. Click on the “H” logo to the left of the field answer options. (See Figure 4. & 5.). The date/time of change, the user and the data changes made are visible. The data history results are sorted from earliest to most recent.

Figure 4.

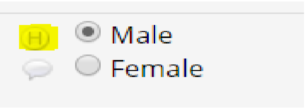

Figure 5.

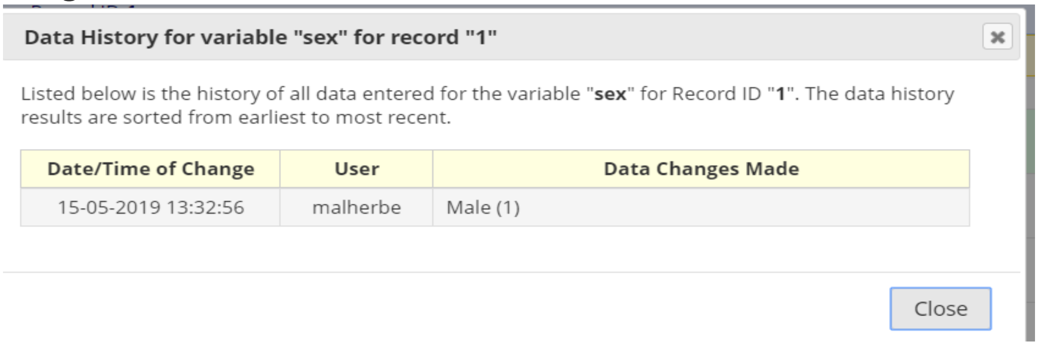

5.9. LOGGING AND AUDIT TRAIL

REDCap maintains a built-in audit trail that logs all user activity and all pages viewed by every user, including contextual information (e.g. the project or record being accessed). Whether the activity be entering data, exporting data, modifying a field, running a report, or add/modifying a user, among a plethora of other activities, REDCap logs all actions. The logging record can itself be viewed within a project by users that have been given privileges to view the Logging page. The Logging page allows such users to view or export the entire audit trail for that project, and also to filter the audit trail in various ways based upon the type of activity and/or user. The built-in audit trail in REDCap allows

administrators to be able to determine all the activity and all the data viewed or modified by any given user.

5.10. SECURITY

REDCap is a web application for building and managing online surveys and databases, and thus proper security practices must be instituted on the network and server(s) hosting REDCap and also within the REDCap software itself. This level of security exists in order to protect the data stored within REDCap as well as information pertaining to the identity and activity of REDCap end-users.

Much of the security surrounding REDCap has nothing to do with the REDCap software itself, but rather it relies on the IT infrastructure and environment in which REDCap has been installed. This includes the web server and database server, as well as the connection between the two and the connection of the web server with the REDCap end-user.

REDCap infrastructure requirements and dependencies:

1) Web server, such as Microsoft IIS or Apache
    a. PHP – version 5.0.0 and up (download and install PHP)
    b. PHP frameworks and extensions needed
        i. PEAR framework must be installed with DB and Auth modules (how to install PEAR)
        ii. cURL extension is required for some optional components (how to install cURL)
2) Database server
    a. MySQL database – version 5.0.0 and up (how to install MySQL)
    b. MySQL client – required for installation/upgrades (e.g. phpMyAdmin, MySQL Workbench)
3) SMTP email server of any kind – must be configured with PHP on your web server; can be installed on the same web server or a separate server (preferred)

An important aspect of security is user privileges. Permission to access REDCap has to be granted by the administrator/Data Managers. He/she will create new REDCap user

accounts using their name, surname, email address and their institution ID. SUN-IRG falls under the institute ID of SU_MBHG. The user will then be issued with a username and password via an automated email. User rights within any given project is outlined in point 5.4. Database User Rights.

REDCap contains an auto-logout setting and will automatically log a user out of the system if they have not had any activity (e.g. typing, moving the mouse) on their current web page for the set amount of time. This prevents someone else from accessing their account and their project data if they leave a workstation without properly logging out or closing their browser window. There exist some customizable settings that govern login activity, such as being able to manually set the number of failed login attempts before a user is locked out of the system for a specified amount of time. Also available is a user suspension status, which can be set for any given user. Suspending a user allows them to remain a user in the system but denying them access to the entire REDCap application until their suspended status has been revoked. For various reasons, suspending a user is preferable to deleting the user permanently from the system.

REDCap Data Storage REDCap stores its data and all system and project information in various relational database tables (i.e. utilizing foreign keys and indexes) within a single MySQL database, which is an open source RDBMS (relational database management system). The front end of REDCap is written in PHP, which is widely used, robust, open

source scripting language. Setting up the web server and database server and securing the communication of the servers to each other and to the end-user are the responsibilities of the partner institution that is installing REDCap, and thus must be completed prior to installing REDCap. The institution installing REDCap will store all data captured in REDCap on its own servers. Thus, all project data is stored and hosted there at the local institution, and no project data is ever transmitted at any time by REDCap from that institution to another institution or organization. REDCap's native webpage encoding and database storage collation is UTF-8, which allows for non-English languages to be utilized in user-defined text that gets stored in REDCap. This includes data entered for a project or the text defined for a survey question or database field label, among many other types of

user-defined text. REDCap's database tables implement MySQL's Innodb storage engine, which allows for the use of foreign keys for referential integrity, transactions, and row-level locking (as opposed to table-level locking), all of which are needed in REDCap for consistency, performance, and scalability.

To help protect and secure the data stored in REDCap's back end database, the software application employs various methods to protect against malicious users who may attempt to identify and exploit any security vulnerabilities in the system. Such methods will be described here in technical detail. In REDCap, all incoming data gets intentionally filtered, sanitized, and escaped. This includes all data submitted in an HTTP Post request and all query string data found in every URL while accessing REDCap, among other modes through which user-defined data gets submitted in the application. Server environment variables that are vulnerable to forgery by users are also checked and sanitized. All user submitted data is properly filtered for any possibly harmful markup tags (e.g. <script>) and is then escaped before ever being displayed on a web page within the application. SQL queries sent to the database server from REDCap are all properly escaped before being sent. If any values used in an SQL query originated from user-defined values, they would have already been sanitized beforehand as well, as described above. User-defined data used within SQL queries also have their data type checked to prevent any mismatching of data types (e.g. making sure a number is really a number). These processes of sanitization, filtering, data type checking, and escaping all help to protect against methods of attack, such as Cross-Site Scripting (XSS) and SQL Injection. To specifically protect against Cross-Site Request Forgery (CSRF), which is another method of attack, REDCap utilizes a "nonce" (a secret, user-specific token) on every web form used in the application. The nonce is generated anew on each web page as the user navigates within REDCap during a session.

5.11. DATA ARCHIVAL

If you are finished with a project (data is cleaned and locked) and wish to make it completely inaccessible, you may mark the project as 'Completed'. Doing so will take it offline and remove it from everyone's project list, after which it can only be seen again by clicking the *Show Completed Projects* link at the bottom of the *My*

Projects page. Once marked as Completed, no one in the project (except for REDCap administrators) can access the project, and only administrators may undo the Completion and return it back to an accessible state for all project users.

5.12. DATA STORAGE & BACK-UP

REDCap project users are responsible for managing and maintaining their own REDCap projects and each project's accompanying data. REDCap Administrators cannot *undo* individual project or data changes made to a project (or to project data) by a research team. The REDCap data on the Hermes server is backed up daily to a secure server in a separate building and back-ups are archived at increasing intervals for a period of 2 years. The data server and web server are separate. Using the Data Export Tool, all the raw data and syntax files from the study are exported and saved as a single .CSV file onto an external hard drive once weekly.

6. DEFINITIONS

None

7. REFERENCES

None

8. SUPPORTING DOCUMENTS

1) Data Resolution Workflow Summary log
2) FIT-001 Data Problem Summary Form
3) Data Quality Rules Discrepancy Log
4) Data Dictionary/Codebook Change Log
5) Results Data Verification Log
6) Missing Results Data Log

9. AVAILABILITY

The original signed version of this document is kept by the Quality Assurance Officer. Training documents available in personnel folders. A copy of the document can be found on the document management system ALFRESCO.

10. DOCUMENT HISTORY

Version No.	Date	Location of Change History	Author/ Reviewer	Approving Official	Date Approved	Next Review Date
1.0		*New document.*	K Stanley / G van der Spuy	L Muller		
