# Supplementary material for: Cascade Immune Mechanisms of Protection against Mycobacterium tuberculosis (IMPAc-TB): study protocol for the Household Contact Study in the Western Cape, South Africa
Source: BMC Infect Dis. 2022 Apr 15;22:381. doi: 10.1186/s12879-022-07349-8 (PMC9012070; doi:10.1186/s12879-022-07349-8)
Supplement: Supplementary file 3 — Additional file 3. Cascade data dictionary. [file 12879_2022_7349_MOESM3_ESM.pdf]

Variable	REDCap Type	SQL Type	Enumeration	CUI	CUI Name
**Distinct Identifier**					
Record ID	text	CHAR	NA	C0681857	individual as study subject
**Physical At Enrollment**					
TB treatment started?	yesno	BOOLEAN	NA	C0041296; C0013216; C1272689	Tuberculosis; Pharmacotherapy; Started
If yes, date started	text	DATE	NA	C1531783	Date treatment started
Lung disease (excl. TB)	radio	BOOLEAN	1, Yes | 0, No	C0024115	Lung diseases
Diabetes	radio	BOOLEAN	1, Yes | 0, No	C0011849	Diabetes Mellitus
Hypertension	radio	BOOLEAN	1, Yes | 0, No	C0020538	Hypertensive disease
Heart disease	radio	BOOLEAN	1, Yes | 0, No	C0018799	Heart Diseases
Kidney disease	radio	BOOLEAN	1, Yes | 0, No	C0022658	Kidney Diseases
Thyroid disease	radio	BOOLEAN	1, Yes | 0, No	C0040128	Thyroid Diseases
Cancer	radio	BOOLEAN	1, Yes | 0, No	CL604836	Cancer (e.g., lung, breast, genitourinary, colon, etc. in some patients)
Psychiatric disorders	radio	BOOLEAN	1, Yes | 0, No	C1718246	Psychiatric or mood diseases or conditions
Hepatitis B	radio	BOOLEAN	1, Yes | 0, No	C0019163	Hepatitis B
Hepatitis C	radio	BOOLEAN	1, Yes | 0, No	C0019196	Hepatitis C
Other liver diseases	radio	BOOLEAN	1, Yes | 0, No	C0023895	Liver diseases
Asthma	radio	BOOLEAN	1, Yes | 0, No	C0004096	Asthma
Gout	radio	BOOLEAN	1, Yes | 0, No	C0018099	Gout
Arthritis	radio	BOOLEAN	1, Yes | 0, No	C0003864	Arthritis
Alcoholism/Excessive alcohol use	radio	BOOLEAN	1, Yes | 0, No	C0455500	H/O: alcoholism
If yes other lung disease, please provide ICD10 code:	text	VARCHAR	ICD10	CL936147	ICD10, 2016
If yes other lung disease, please provide ICD10 code:	text	VARCHAR	ICD10	CL936147	ICD10, 2016
If yes other lung disease, please provide ICD10 code:	text	VARCHAR	ICD10	CL936147	ICD10, 2016
If yes diabetes, please provide ICD10 code:	text	VARCHAR	ICD10	CL936147	ICD10, 2016
If yes diabetes, please provide ICD10 code:	text	VARCHAR	ICD10	CL936147	ICD10, 2016
If yes diabetes, please provide ICD10 code:	text	VARCHAR	ICD10	CL936147	ICD10, 2016
If yes hypertension, please provide ICD10 code:	text	VARCHAR	ICD10	CL936147	ICD10, 2016
If yes hypertension, please provide ICD10 code:	text	VARCHAR	ICD10	CL936147	ICD10, 2016
If yes hypertension, please provide ICD10 code:	text	VARCHAR	ICD10	CL936147	ICD10, 2016

Variable	REDCap Type	SQL Type	Enumeration	CUI	CUI Name
If yes heart disease, please provide ICD10 code:	text	VARCHAR	ICD10	CL936147	ICD10, 2016
If yes heart disease, please provide ICD10 code:	text	VARCHAR	ICD10	CL936147	ICD10, 2016
If yes heart disease, please provide ICD10 code:	text	VARCHAR	ICD10	CL936147	ICD10, 2016
If yes kidney disease, please provide ICD10 code:	text	VARCHAR	ICD10	CL936147	ICD10, 2016
If yes kidney disease, please provide ICD10 code:	text	VARCHAR	ICD10	CL936147	ICD10, 2016
If yes kidney disease, please provide ICD10 code:	text	VARCHAR	ICD10	CL936147	ICD10, 2016
If yes thyroid disease, please provide ICD10 code:	text	VARCHAR	ICD10	CL936147	ICD10, 2016
If yes thyroid disease, please provide ICD10 code:	text	VARCHAR	ICD10	CL936147	ICD10, 2016
If yes thyroid disease, please provide ICD10 code:	text	VARCHAR	ICD10	CL936147	ICD10, 2016
If yes cancer, please provide ICD10 code:	text	VARCHAR	ICD10	CL936147	ICD10, 2016
If yes cancer, please provide ICD10 code:	text	VARCHAR	ICD10	CL936147	ICD10, 2016
If yes cancer, please provide ICD10 code:	text	VARCHAR	ICD10	CL936147	ICD10, 2016
If yes psychiatric disorder, please provide ICD10 code:	text	VARCHAR	ICD10	CL936147	ICD10, 2016
If yes psychiatric disorder, please provide ICD10 code:	text	VARCHAR	ICD10	CL936147	ICD10, 2016
If yes psychiatric disorder, please provide ICD10 code:	text	VARCHAR	ICD10	CL936147	ICD10, 2016
If yes Hep B, please provide ICD10 code:	text	VARCHAR	ICD10	CL936147	ICD10, 2016
If yes Hep B, please provide ICD10 code:	text	VARCHAR	ICD10	CL936147	ICD10, 2016
If yes Hep B, please provide ICD10 code:	text	VARCHAR	ICD10	CL936147	ICD10, 2016
If yes Hep C, please provide ICD10 code:	text	VARCHAR	ICD10	CL936147	ICD10, 2016
If yes Hep C, please provide ICD10 code:	text	VARCHAR	ICD10	CL936147	ICD10, 2016
If yes Hep C, please provide ICD10 code:	text	VARCHAR	ICD10	CL936147	ICD10, 2016
If yes other liver disease, please provide ICD10 code:	text	VARCHAR	ICD10	CL936147	ICD10, 2016
If yes other liver disease, please provide ICD10 code:	text	VARCHAR	ICD10	CL936147	ICD10, 2016
If yes other liver disease, please provide ICD10 code:	text	VARCHAR	ICD10	CL936147	ICD10, 2016
If yes asthma, please provide ICD10 code:	text	VARCHAR	ICD10	CL936147	ICD10, 2016

Variable	REDCap Type	SQL Type	Enumeration	CUI	CUI Name
If yes asthma, please provide ICD10 code:	text	VARCHAR	ICD10	CL936147	ICD10, 2016
If yes asthma, please provide ICD10 code:	text	VARCHAR	ICD10	CL936147	ICD10, 2016
If yes gout, please provide ICD10 code:	text	VARCHAR	ICD10	CL936147	ICD10, 2016
If yes gout, please provide ICD10 code:	text	VARCHAR	ICD10	CL936147	ICD10, 2016
If yes gout, please provide ICD10 code:	text	VARCHAR	ICD10	CL936147	ICD10, 2016
If yes arthritis, please provide ICD10 code:	text	VARCHAR	ICD10	CL936147	ICD10, 2016
If yes arthritis, please provide ICD10 code:	text	VARCHAR	ICD10	CL936147	ICD10, 2016
If yes arthritis, please provide ICD10 code:	text	VARCHAR	ICD10	CL936147	ICD10, 2016
If yes alcoholism, please provide ICD10 code:	text	VARCHAR	ICD10	CL936147	ICD10, 2016
If yes alcoholism, please provide ICD10 code:	text	VARCHAR	ICD10	CL936147	ICD10, 2016
If yes alcoholism, please provide ICD10 code:	text	VARCHAR	ICD10	CL936147	ICD10, 2016
**Smoking History**					
Has the participant ever smoked tobacco?	radio	ENUMERATE	1, Yes | 0, No | 98, Unknown	C0543414	Tobacco use
If yes, is the smoking ongoing?	radio	ENUMERATE	1, Yes | 0, No | 98, Unknown	C3853727	Tobacco user
Duration of smoking (current/past):	text	INT	NA	C0543414; C0449238	Tobacco use; Duration (temporal concept)
Duration unit:	radio	ENUMERATE	1, Months | 2, Years	C1519384; C0449238	Tobacco Smoking History; Duration (temporal concept)
Has participant quit smoking?	radio	ENUMERATE	1, Yes | 0, No | 98, Unknown	C1698618	Ex-tobacco user
How long since quitting?	text	INT	NA	C1698618; C1707889	Ex-tobacco user; Elapse
Duration unit:	radio	ENUMERATE	1, Months | 2, Years	C1519384; C0449238	Tobacco Smoking History; Duration (temporal concept)
**Tuberculosis History**					
Any previous TB episodes?	radio	ENUMERATE	1, Yes | 0, No | 2, Unknown	C0041296; C3258978	Tuberculosis; Medical History
Year of last TB episode?	text	VARCHAR	NA	C0041296; C3258978	Tuberculosis; Medical History
Number of previously treated TB episodes:	text	VARCHAR	NA	C0041296; C3258978	Tuberculosis; Medical History
Was one of those previous episodes DRTB?	radio	ENUMERATE	1, Yes | 0, No | 98, Unknown	C0041296; C3258978	Tuberculosis; Medical History
When was most recent TB treatment completed?	radio	ENUMERATE	1, One year ago | 2, >= one year ago	C0040223	Time, temporal concept
History of BCG vaccination	radio	ENUMERATE	1, Yes | 0, No | 98, Unknown	C3257574; C3258978	BCG Vaccine; Medical History
Does the participant report any of the following symptoms listed below?	yesno	BOOLEAN	NA	C3258978	Medical History
Night sweats	radio	ENUMERATE	1, Less than one week | 2, At least 1 week and less than 2 weeks | 3, At least 2 weeks | 0, None	C0028081; C3258978; C0449238	Night sweats; Medical History; Duration, temporal concept
Fevers	radio	ENUMERATE	1, Less than one week | 2, At least 1 week and less than 2 weeks | 3, At least 2 weeks | 0, None	C0015967; C3258978; C0449238	Fevers; Medical History; Duration, temporal concept

Variable	REDCap Type	SQL Type	Enumeration	CUI	CUI Name
Loss of appetite	radio	ENUMERATE	1, Less than one week | 2, At least 1 week and less than 2 weeks | 3, At least 2 weeks | 0, None	C0232462; C3258978; C0449238	Decrease in appetite; Medical History; Duration, temporal concept
Unintentional Weight loss	radio	ENUMERATE	1, Less than one week | 2, At least 1 week and less than 2 weeks | 3, At least 2 weeks | 0, None	C0936227; C3258978; C0449238	Abnormal weight loss (finding); Medical History; Duration, temporal concept
Malaise/fatigue	radio	ENUMERATE	1, Less than one week | 2, At least 1 week and less than 2 weeks | 3, At least 2 weeks | 0, None	C0024528; C3258978; C0449238	Malaise and fatigue; Medical History; Duration, temporal concept
Cough	radio	ENUMERATE	1, Less than one week | 2, At least 1 week and less than 2 weeks | 3, At least 2 weeks | 0, None	C3258978; C0449238	Medical History; Duration, temporal concept
Sputum production	radio	ENUMERATE	1, Less than one week | 2, At least 1 week and less than 2 weeks | 3, At least 2 weeks | 0, None	C3258978; C0449238	Medical History; Duration, temporal concept
Haemoptysis	radio	ENUMERATE	1, Less than one week | 2, At least 1 week and less than 2 weeks | 3, At least 2 weeks | 0, None	C3258978; C0449238	Medical History; Duration, temporal concept
Dyspnoea	radio	ENUMERATE	1, Less than one week | 2, At least 1 week and less than 2 weeks | 3, At least 2 weeks | 0, None	C3258978; C0449238	Medical History; Duration, temporal concept
Pleuritic chest pain	radio	ENUMERATE	1, Less than one week | 2, At least 1 week and less than 2 weeks | 3, At least 2 weeks | 0, None	C3258978; C0449238	Medical History; Duration, temporal concept
Abdominal pain	radio	ENUMERATE	1, Less than one week | 2, At least 1 week and less than 2 weeks | 3, At least 2 weeks | 0, None	C3258978; C0449238	Medical History; Duration, temporal concept
Nausea	radio	ENUMERATE	1, Less than one week | 2, At least 1 week and less than 2 weeks | 3, At least 2 weeks | 0, None	C3258978; C0449238	Medical History; Duration, temporal concept
Vomiting	radio	ENUMERATE	1, Less than one week | 2, At least 1 week and less than 2 weeks | 3, At least 2 weeks | 0, None	C3258978; C0449238	Medical History; Duration, temporal concept
**Medications History**					
Is the participant currently on medication? If yes, please continue with name of drug,dose etc.	yesno	BOOLEAN	NA	CL972932	History of medication
Has the participant taken any medication in past 8 weeks? If yes, please continue with name of drug,dose etc.	yesno	BOOLEAN	NA	CL972932	History of medication
Drug name #1:	text	VARCHAR	NA	NULL	Translate to RxNorm post facto
Drug name #2:	text	VARCHAR	NA	NULL	Translate to RxNorm post facto
Drug name #3:	text	VARCHAR	NA	NULL	Translate to RxNorm post facto
Drug name #4:	text	VARCHAR	NA	NULL	Translate to RxNorm post facto

Variable	REDCap Type	SQL Type	Enumeration	CUI	CUI Name
Drug name #5:	text	VARCHAR	NA	NULL	Translate to RxNorm post facto
Drug name #6:	text	VARCHAR	NA	NULL	Translate to RxNorm post facto
Drug name #1:	text	VARCHAR	NA	NULL	Translate to RxNorm post facto
Drug name #2:	text	VARCHAR	NA	NULL	Translate to RxNorm post facto
Drug name #3:	text	VARCHAR	NA	NULL	Translate to RxNorm post facto
Drug name #4:	text	VARCHAR	NA	NULL	Translate to RxNorm post facto
Drug name #5:	text	VARCHAR	NA	NULL	Translate to RxNorm post facto
Drug name #6:	text	VARCHAR	NA	NULL	Translate to RxNorm post facto
**PET/Bronchoscopy Physical**					
CD4 cell count result done & available	radio	ENUMERATE	1, Yes | 0, No | 99, N/A	CL523893	CD4 Count determination procedure
CD4 cell count [cells/mm3]	text	INT	NA	C3541261	CD4 Expressing Cell Count
HIV viral load result done & available	radio	ENUMERATE	1, Yes | 0, No | 99, N/A	C1168369	HIV Viral Load Measurement
HIV viral load [copies/ml]	text	INT	NA	C1168369; C0456984	HIV Viral Load Measurement; Test Result
HIV ELISA test result	radio	ENUMERATE	0, Negative | 1, Positive | 98, Not Done	C0201388; C0456984	HIV-1 ELISA assay; Test Result
Weight (kg)	text	INT	NA	C0005910; C0031809	Body Weight; Physical Examination
Height (cm)	text	INT	NA	C0489786; C0031809	Body Height; Physical Examination
Blood Pressure: systolic	text	INT	NA	C0871470; C0031809	Systolic Blood Pressure; Physical Examination
Repeat Blood Pressure: systolic	text	INT	NA	C0871470; C0031809	Systolic Blood Pressure; Physical Examination
Blood Pressure: diastolic	text	INT	NA	C0428883; C0031809	Diastolic Blood Pressure; Physical Examination
Repeat Blood Pressure: diastolic	text	INT	NA	C0428883; C0031809	Diastolic Blood Pressure; Physical Examination
Temperature (?C)	text	DOUBLE	NA	C0005903; C0031809	Body Temperature; Physical Examination
Pulse Rate	text	INT	NA	C0232117; C0031809	Pulse Rate; Physical Examination
Respiratory rate/min	text	INT	NA	C0231832	Respiratory Rate
Hb [g/dL]	text	DOUBLE	NA	C1984226	Hemoglobin test status
Random HGT [mmol/L]	text	DOUBLE	NA	C0428567	Random blood glucose measurement
HbA1c result	text	DOUBLE	NA	C0474680	Hemoglobin A1c measurement
**Chest X-Ray**					
Film quality?	radio	ENUMERATE	1, Optimal | 2, Sub-optimal | 3, Unreadable	C0806487	Image Quality
Infiltrate or Consolidation	checkbox	ENUMERATE	1, Infiltrate or Consolidation	NULL	
Any cavity lesion	checkbox	ENUMERATE	2, Any cavity lesion	NULL	
Nodule with poorly defined margins	checkbox	ENUMERATE	3, Nodule with poorly defined margins	NULL	

Variable	REDCap Type	SQL Type	Enumeration	CUI	CUI Name
Pleural effusion	checkbox	ENUMERATE	4, Pleural effusion	NULL	
Hilar or Mediastinal Lymphadenopathy	checkbox	ENUMERATE	5, Hilar or Mediastinal Lymphadenopathy	NULL	
Other findings suggestive of TB e.g. milliary TB	checkbox	ENUMERATE	6, Other findings suggestive of TB e.g. milliary TB	NULL	
Right Upper Lobe	checkbox	ENUMERATE	1, Right Upper Lobe	C1261074	Upper lobe of the right lung
Right Middle Lobe	checkbox	ENUMERATE	2, Right Middle Lobe	C0225757	Middle lobe of the right lung
Right Lower Lobe	checkbox	ENUMERATE	3, Right Lower Lobe	C1261075	Lower lobe of the right lung
Left Upper Lobe	checkbox	ENUMERATE	4, Left Upper Lobe	C1261076	Upper lobe of left lung
Left Lower Lobe	checkbox	ENUMERATE	5, Left Lower Lobe	C1261077	Lower lobe of left lung
Modified Ralph score:	text	DOUBLE	NA	NULL	
**Inclusion Criteria**					
Age 18 -65 years	radio	ENUMERATE	1, Yes | 0, No | 99, Not Applicable	C1704756; C0001779	Trial Inclusion and Exclusion Criteria Domain; Age
Weight >35kg and < 120kg	radio	ENUMERATE	1, Yes | 0, No | 99, Not Applicable	C1704756; C0005910	Trial Inclusion and Exclusion Criteria Domain; Body Weight
Absence of current source of infection	radio	ENUMERATE	1, Yes | 0, No | 99, Not Applicable	C1704756; C3714514	Trial Inclusion and Exclusion Criteria Domain; Infection
HIV negative	radio	ENUMERATE	1, Yes | 0, No | 99, Not Applicable	C1704756; C0458074	Trial Inclusion and Exclusion Criteria Domain; HIV status
Newly diagnosed, recurrent TB with previous TB treatment completed at least 36 months prior to recruitment.	radio	ENUMERATE	1, Yes | 0, No | 99, Not applicable	C1704756; C0857427	Trial Inclusion and Exclusion Criteria Domain; Old Tuberculosis
Rifampicin sensitivity confirmed by Gene Xpert	radio	ENUMERATE	1, Yes | 0, No | 99, Not applicable	C1704756; C0035608; C0025948	Trial Inclusion and Exclusion Criteria Domain; Rifampin; Microbial Sensitivity Test
No TB in past 3 years (this 3 years start when the tx is done)	radio	ENUMERATE	1, Yes | 0, No | 99, Not applicable	C1704756; C0857427	Trial Inclusion and Exclusion Criteria Domain; Old Tuberculosis
They are smear positive OR Xpert positive (medium/high or very high) OR Xpert positive lower than medium with either symptoms or CXR positive or active TB	radio	ENUMERATE	1, Yes | 0, No | 99, Not applicable	ICD10-A15.0; ICD10-A15.3; C0039985	Tuberculosis of lung, confirmed by sputum microscopy with or without culture;
Absent history of TB or known exposure to a TB patient	radio	ENUMERATE	1, Yes | 0, No | 99, Not applicable	C1704756; C0857427; C1708498	Trial Inclusion and Exclusion Criteria Domain; Old Tuberculosis; Indirect Contact Transmission Infection; Tuberculosis of lung, confirmed by unspecified means; Chest Radiography
HIV negative	radio	ENUMERATE	1, Yes | 0, No | 99, Not applicable	C1704756; C0458074	Trial Inclusion and Exclusion Criteria Domain; HIV status
No TB for 3 years (This 3 yrs start when the treatment is done)	radio	ENUMERATE	1, Yes | 0, No | 99, Not applicable	C1704756; C0857427	Trial Inclusion and Exclusion Criteria Domain; Old Tuberculosis
Xpert negative	radio	ENUMERATE	1, Yes | 0, No | 99, Not applicable	C0587081	Laboratory Test Result
No symptoms/CXR signs	radio	ENUMERATE	1, Yes | 0, No | 99, Not applicable	C0039985	Chest Radiography
No known contact for past 1 yr	radio	ENUMERATE	1, Yes | 0, No | 99, Not applicable	NULL	

Variable	REDCap Type	SQL Type	Enumeration	CUI	CUI Name
**Study Arm**					
Assigned to Arm 3: Comm. Ctls (HN)	yesno	BOOLEAN	NA	NULL	
Assigned to Arm 2: TB Cases (HN)	yesno	BOOLEAN	NA	NULL	
Assigned to Arm 4: Close Contacts (HN)	yesno	BOOLEAN	NA	NULL	
Assigned to Arm 5: HIV Positive	yesno	BOOLEAN	NA	NULL	
**Laboratory GeneXpert**					
From where was the sputum collected that yielded this GeneXpert result?	radio	ENUMERATE	1, Research sputum collection | 2, Routine clinical care at treating facility | 3, No GeneXpert was done	NULL	
GeneXpert date done?	text	DATE	NA	C0011008	Date, temporal concept
Positive result found?	radio	ENUMERATE	1, Yes | 0, No | 99, Result not available	C0587081	Laboratory Test Result
Rif resistance found?	radio	ENUMERATE	1, Yes | 0, No | 99, Result not available	C0035608; C0025948	Rifampin; Microbial Sensitivity Test
Xpert ranking?	radio	ENUMERATE	1, Very low | 2, Low | 3, Medium | 4, High | 5, Very high | 99, Not available	NULL	
Where was this repeat done?	radio	ENUMERATE	1, Research lab | 2, Reference lab | 3, No GeneXpert repeat was done	NULL	
GeneXpert date done?	text	DATE	NA	C0011008	Date, temporal concept
Positive result found?	radio	ENUMERATE	1, Yes | 0, No | 99, Result not available	C0587081	Laboratory Test Result
Rif resistance found?	radio	ENUMERATE	1, Yes | 0, No | 99, Result not available	C0035608; C0025948	Rifampin; Microbial Sensitivity Test
Xpert ranking?	radio	ENUMERATE	1, Very low | 2, Low | 3, Medium | 4, High | 5, Very high | 99, Not available	NULL	
**PET Result**					
Visible lesions on CT:	yesno	BOOLEAN	NA	C0202823	Chest CT
If yes, how many?	dropdown	ENUMERATE	1, 1 | 2, 2 | 3, 3 | 4, 4	NULL	
Is the lesion in keeping with TB lesion?	yesno	BOOLEAN	NA	NULL	
Main location of lesions:	radio	ENUMERATE	1, LUL | 2, RUL | 3, RML | 4, LLL | 5, RLL	C1261076; C1261074; C0225757; C1261077; C1261075	Upper lobe of left lung; Upper lobe of the right lung; Middle lobe of the right lung; Lower lobe of left lung; Lower lobe of the right lung
Morphology of lesions:	dropdown	ENUMERATE	1, Enlarged lymphnode | 2, Nodule | 3, Consolidation | 4, Cavity | 5, Tree-in-bud | 6, Bronchial thickening | 7, Linear scar | 8, Fibrosis | 9, Bronchiectasis	NULL	

Variable	REDCap Type	SQL Type	Enumeration	CUI	CUI Name
			(ground-glass) | 10, Mass | 11, Bulla(e) | 12, Other		
If other, comment	text	VARCHAR	NA	NULL	
Does the lesion have increased FDG-avidity?	yesno	BOOLEAN	NA	NULL	
If yes, SUVmax:	text	DOUBLE	NA	NULL	
If yes, metabolic lesion volume:	text	DOUBLE	NA	NULL	
If yes, total glycolytic activity:	text	DOUBLE	NA	NULL	
Is the lesion in keeping with TB lesion?	yesno	BOOLEAN	NA	NULL	
Main location of lesions:	radio	ENUMERATE	1, LUL | 2, RUL | 3, RML | 4, LLL | 5, RLL	C1261076; C1261074; C0225757; C1261077; C1261075	Upper lobe of left lung; Upper lobe of the right lung; Middle lobe of the right lung; Lower lobe of left lung; Lower lobe of the right lung
Morphology of lesions:	dropdown	ENUMERATE	1, Enlarged lymphnode | 2, Nodule | 3, Consolidation | 4, Cavity | 5, Tree-in-bud | 6, Bronchial thickening | 7, Linear scar | 8, Fibrosis | 9, Bronchiectasis (ground-glass) | 10, Mass | 11, Bulla(e) | 12, Other	NULL	
If other, comment	text	VARCHAR	NA	NULL	
Does the lesion have increased FDG-avidity?	yesno	BOOLEAN	NA	NULL	
If yes, SUVmax:	text	DOUBLE	NA	NULL	
If yes, metabolic lesion volume:	text	DOUBLE	NA	NULL	
If yes, total glycolytic activity:	text	DOUBLE	NA	NULL	
Is the lesion in keeping with TB lesion?	yesno	BOOLEAN	NA	NULL	
Main location of lesions:	radio	ENUMERATE	1, LUL | 2, RUL | 3, RML | 4, LLL | 5, RLL	C1261076; C1261074; C0225757; C1261077; C1261075	Upper lobe of left lung; Upper lobe of the right lung; Middle lobe of the right lung; Lower lobe of left lung; Lower lobe of the right lung
Morphology of lesions:	dropdown	ENUMERATE	1, Enlarged lymphnode | 2, Nodule | 3, Consolidation | 4, Cavity | 5, Tree-in-bud | 6, Bronchial thickening | 7, Linear scar | 8, Fibrosis | 9, Bronchiectasis (ground-glass) | 10, Mass | 11, Bulla(e) | 12, Other	NULL	
If other, comment	text	VARCHAR	NA	NULL	
Does the lesion have increased FDG-avidity?	yesno	BOOLEAN	NA	NULL	
If yes, SUVmax:	text	DOUBLE	NA	NULL	
If yes, metabolic lesion volume (ml):	text	DOUBLE	NA	NULL	

	Variable	REDCap Type	SQL Type	Enumeration	CUI	CUI Name
	If yes, total glycolytic activity:	text	DOUBLE	NA	NULL	
	Is the lesion in keeping with TB lesion?	yesno	BOOLEAN	NA	NULL	
	Main location of lesions:	radio	ENUMERATE	1, LUL | 2, RUL | 3, RML | 4, LLL | 5, RLL	C1261076; C1261074; C0225757; C1261077; C1261075	
	Morphology of lesions:	dropdown	ENUMERATE	1, Enlarged lymphnode | 2, Nodule | 3, Consolidation | 4, Cavity | 5, Tree-in-bud | 6, Bronchial thickening | 7, Linear scar | 8, Fibrosis | 9, Bronchiectasis (ground-glass) | 10, Mass | 11, Bulla(e) | 12, Other	NULL	
	If other, comment	text	VARCHAR	NA	NULL	
	Does the lesion have increased FDG-avidity?	yesno	BOOLEAN	NA	NULL	
	If yes, SUVmax:	text	DOUBLE	NA	NULL	
	If yes, metabolic lesion volume:	text	DOUBLE	NA	NULL	
	If yes, total glycolytic activity:	text	DOUBLE	NA	NULL	
**Laboratory** BAL						
	Date BAL sample taken:	text	DATE	NA	C0011008	Date, temporal concept
	Lobe	text	CHAR	NA	C1261076; C1261074; C0225757; C1261077; C1261075	Upper lobe of left lung; Upper lobe of the right lung; Middle lobe of the right lung; Lower lobe of left lung; Lower lobe of the right lung
**Laboratory Quantiferon**						
	QFN GOLD PLUS result?	radio	ENUMERATE	1, Positive | 0, Negative | 2, Indeterminate | 3, Borderline | 98, Not tested	C0587081	Laboratory Test Result
	QFN Nil OD value [IU/ml] {cut-off = < 0.2}	text	DOUBLE	NA	C0587081	Laboratory Test Result
	QFN TB1 OD value [IU/ml] {cut-off = 0.2 - 0.7}	text	DOUBLE	NA	C0587081	Laboratory Test Result
	QFN TB2 OD value [IU/ml] {cut-off = 0.2 - 0.7}	text	DOUBLE	NA	C0587081	Laboratory Test Result
	QFN Mitogen OD value [IU/ml] {cut-off = >0.7}	text	DOUBLE	NA	C0587081	Laboratory Test Result
	QFN TB1 - Nil OD value [IU/ml]	text	DOUBLE	NA	C0587081	Laboratory Test Result
	QFN TB2 - Nil OD value [IU/ml]	text	DOUBLE	NA	C0587081	Laboratory Test Result
	QFN Mitogen - Nil OD value [IU/ml]	text	DOUBLE	NA	C0587081	Laboratory Test Result
	Date QFN ELISA done	text	DATE	NA	C0011008	Date, temporal concept
**Laboratory Sputum**						
	Where is the smear result 1 for this participant from?	radio	ENUMERATE	1, Research smear | 2, Routine clinical care at treating facility | 3, Not Done	NULL	

Variable	REDCap Type	SQL Type	Enumeration	CUI	CUI Name
Smear1 specimen collection date?	text	DATE	NA	C0011008	Date, temporal concept
Smear1 results as pos/neg	radio	ENUMERATE	1, Positive | 0, Negative | 89, Data not known | 99, Not tested	NULL	
Smear1 result as score?	radio	ENUMERATE	0, Negative | 1, 1+ = less than 1 AFB per field | 2, 2+ = 1 to 10 AFB per field | 3, 3+ = more than 10 AFB per field | 4, Less than 10 AFB per all fields | 89, Not available | 99, Not tested	C0587081; C0152616	Laboratory Test Result; Pulmonary tuberculosis, unspecified, tubercle bacilli found (in sputum) by microscopy
Where is the smear result 2 for this participant from?	radio	ENUMERATE	1, Research smear | 2, Routine clinical care at treating facility | 3, Not Done	NULL	
Smear2 specimen collection date?	text	DATE	NA	C0011008	Date, temporal concept
Smear2 results as pos/neg	radio	ENUMERATE	1, Positive | 0, Negative | 89, Data not known | 99, Not tested	C0587081; C0152616	Laboratory Test Result; Pulmonary tuberculosis, unspecified, tubercle bacilli found (in sputum) by microscopy
Smear2 as score?	radio	ENUMERATE	0, Negative | 1, 1+ = less than 1 AFB per field | 2, 2+ = 1 to 10 AFB per field | 3, 3+ = more than 10 AFB per field | 4, Less than 10 AFB per all fields | 89, Not available | 99, Not tested	NULL	
**Laboratory Culture**					
Liquid culture result?	radio	ENUMERATE	1, Positive | 0, Negative | 2, Contaminated | 89, Data not known | 99, Not tested	C0587081	Laboratory Test Result
Date positive?	text	DATE	NA	C0011008	Date, temporal concept
If positive or contaminated, time to positive?	text	INT	NA	C0587081;	Laboratory Test Result;
Time to positive units?	radio	ENUMERATE	1, Hours | 2, Days	C0449238	Duration, temporal concept
Liquid culture Mycobacterium speciation?	dropdown	ENUMERATE	1, M. tuberculosis | 2, M. bovis | 3, M. avium | 4, M. intracellulare | 6, M. kansasii | 7, M. fortuitum | 8, M. asiaticum | 9, Mtb Complex | 30, MOTT, other | 60, Myco spp unknown | 90, Not detected | 99, Not tested	C0587081	Laboratory Test Result
Speciation method?	radio	ENUMERATE	1, Capilia | 2, PCR	C0587081	Laboratory Test Result
ZN of MGIT culture Result1?	radio	ENUMERATE	0, Negative | 1, Negative (contaminated) | 2, Positive | 3, Positive (contaminated) | 99, Not Done	C0587081	Laboratory Test Result

Variable	REDCap Type	SQL Type	Enumeration	CUI	CUI Name
Blood Agar Result1?	radio	ENUMERATE	0, No growth | 1, Growth (contaminants) | 99, Not Done	C0587081	Laboratory Test Result
Mycobacterium Selectatab	radio	ENUMERATE	0, No growth | 1, Growth | 2, Contaminated | 98, Not Done	C0587081	Laboratory Test Result
ZN of Selectatab	radio	ENUMERATE	0, Negative | 1, Negative (contaminated) | 2, Positive | 3, Positive (contaminated) | 99, Not Done	C0587081	Laboratory Test Result
**Study Arm**					
Participant qualifies for the following group:	radio	ENUMERATE	1, Group 4 - Resister (n = 20) | 2, Group 5 - Convertor (n = 20) | 3, Group 6, Reverter (n = 20) | 4, Group 7, IGRA pos, PET neg Adaptive Immune Control (n = 20) | 5, Group 8, IGRA pos, PET pos Subclinical disease (n = 20)	NULL	
Participant qualifies for the following group:	radio	ENUMERATE	1, Group 2 - IGRA neg Community Control (n=20) | 2, Group 3 - IGRA pos Community Control (n =20)	NULL	
PTB confirmed?	yesno	BOOLEAN	NA	C1407689	Primary respiratory tuberculosis
If yes: select all applicable (choice=GeneXpert positive)	checkbox	ENUMERATE	1, GeneXpert positive	C1407689; C1880302	Primary respiratory tuberculosis; Diagnostic Laboratory Biomarker Analysis
If yes: select all applicable (choice=Smear positive)	checkbox	ENUMERATE	2, Smear positive	C0152548	Primary tuberculous infection, unspecified type, tubercle bacilli found (in sputum) by microscopy
If yes: select all applicable (choice=Culture positive)	checkbox	ENUMERATE	3, Culture positive	C0858489	Primary tuberculous infection, unspecified type, confirmed by bacterial culture
If yes: select all applicable (choice=CXR suggest active TB)	checkbox	ENUMERATE	4, CXR suggest active TB	C1407689; C0039985	Primary respiratory tuberculosis; Chest Radiography
